# Supplementary material for: PCBP1 orchestrates amino acid metabolism burst during the naïve-to-primed pluripotency transition
Source: Stem Cell Reports. 2026 Jun 4;21(7):102950. doi: 10.1016/j.stemcr.2026.102950 (PMC13385435; doi:10.1016/j.stemcr.2026.102950)
Supplement: Document S1. Figures S1–S4 and Tables S1–S4 [file mmc1.pdf]

Stem Cell Reports, Volume *21*

Supplemental Information

PCBP1 orchestrates amino acid metabolism burst during the naïve-to-primed pluripotency transition

Evgeny I. Bakhmet, Evgeniy V. Potapenko, Oleg Y. Shuvalov, Arseniy A. Lobov, Egor A. Repkin, Daria V. Kriger, Nadezhda E. Vorobyeva, Alexey N. Korablev, Anna S. Zinovyeva, Andrey A. Kuzmin, Nikolay D. Aksenov, Arthur T. Kopylov, Guangming Wu, Hans R. Schöler, and Alexey N. Tomilin

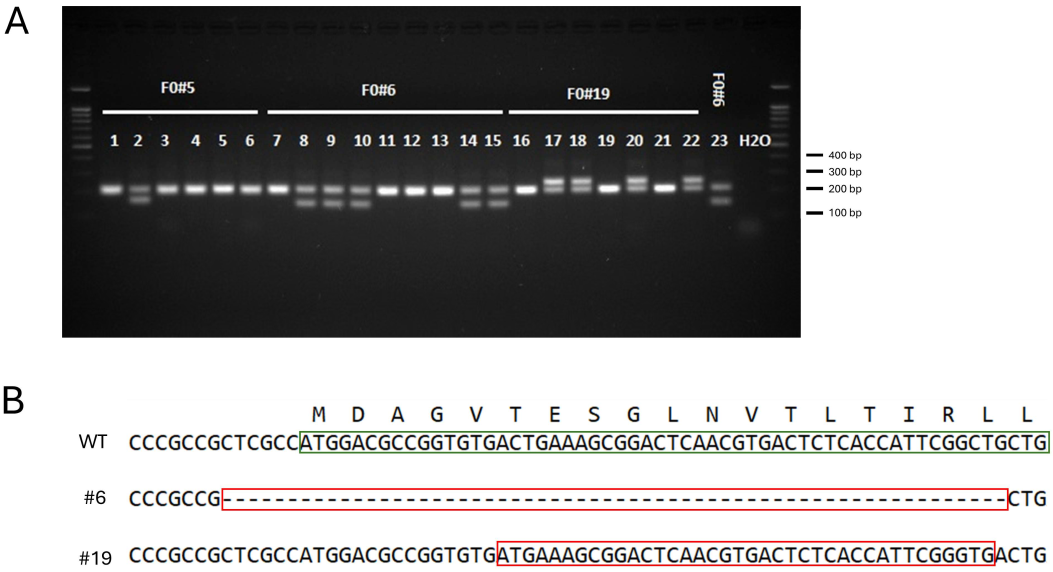

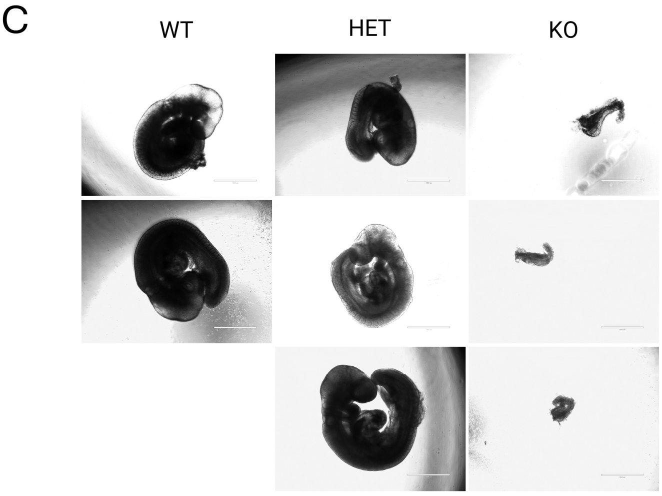

Stage	Decidua	WT	HET	KO
E6.5	7	2	2	2
E7.5	10	2	4	3
E8.5	11	2	6	2
E9.5	10	2	5	3

Figure S1. Heterozygous *Pcbp1*-null embryos characterization

(A) Genotyping of the F0 offspring reveals wild-type and heterozygous pups with deletions (#5 and #6) and an insertion #19 at the start of the protein-coding sequence of the *Pcbp1* gene. F1 offspring from the #6 and #19 founders were selected for further analysis and mice colony propagation. (B) Alignments showing affected alleles of the #6 (60 bp deletion) and #19 (38 bp insertion) lines. (C) Left panel: microphotographs of E9.5 embryos after intercrossing of *Pcbp1*-heterozygous mice. Scale bar: 1000 µm. Right panel: table representing number of decidua and recovered embryos at different developmental stages.

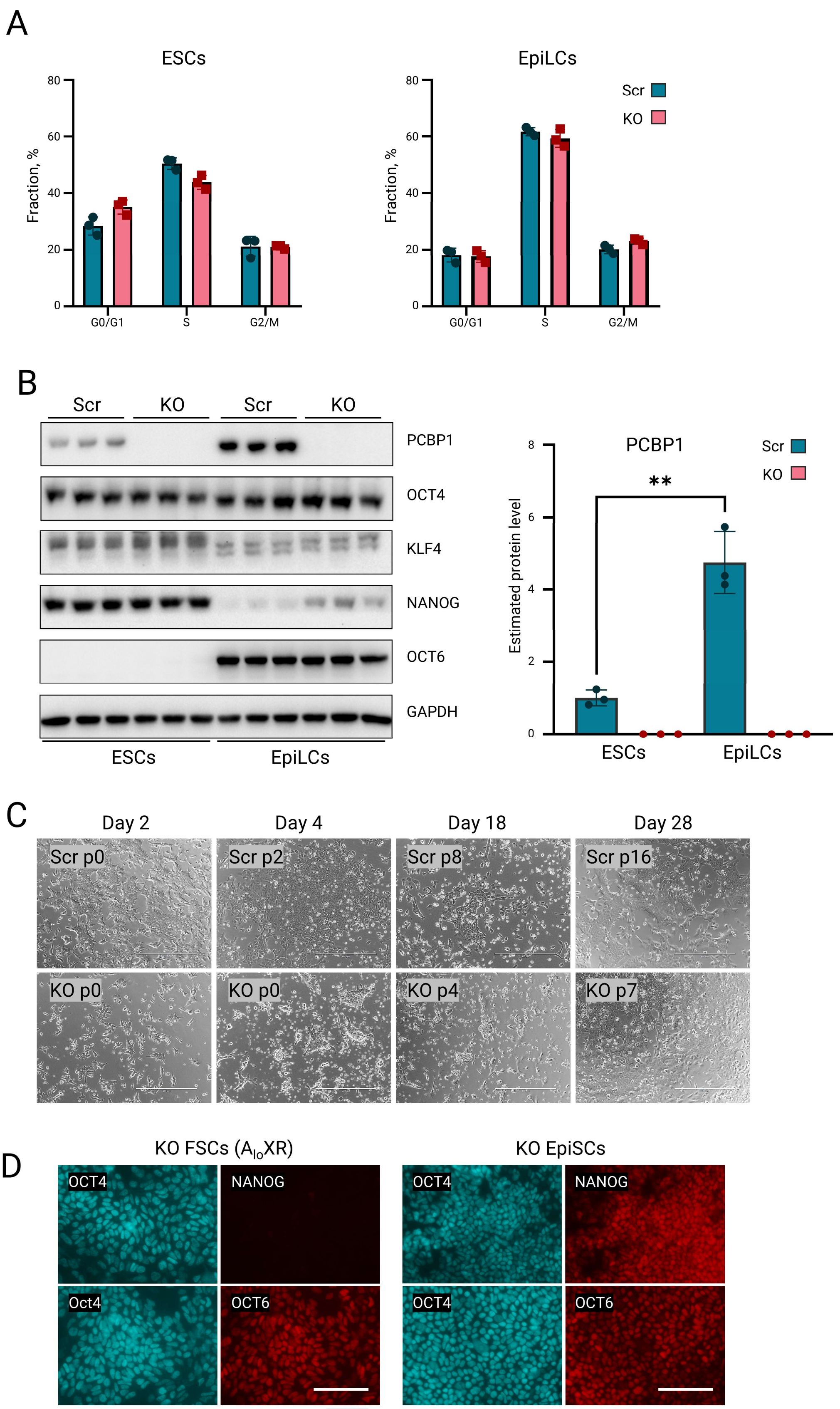

Figure S2. Naïve-to-Primed pluripotency transition of the KO ESCs

(A) Cell cycle analysis of Scr and KO cells in the naïve (ESC) and formative (EpiLC) states of pluripotency. N = 3 biological replicates (individual cell clones); error bars indicate SD of the mean. (B) Left panel: western blot

analysis of Scr and KO cells in ESC and EpiLC states for pluripotency markers. Right panel: quantification analysis of PCBP1 amount from western blot. N = 3 biological replicates (individual cell clones). Error bars indicate SD of the mean. ** $p < 0.01$. (C) Microphotographs of Scr and KO cells passaged in formative $A_{lo}XR$ culture medium. Scale bar: 400 µm. (D) Immunofluorescence microscopy of *Pcbp1*-KO cells after passaging for 28 days in $A_{lo}XR$ medium. Scale bar: 100 µm.

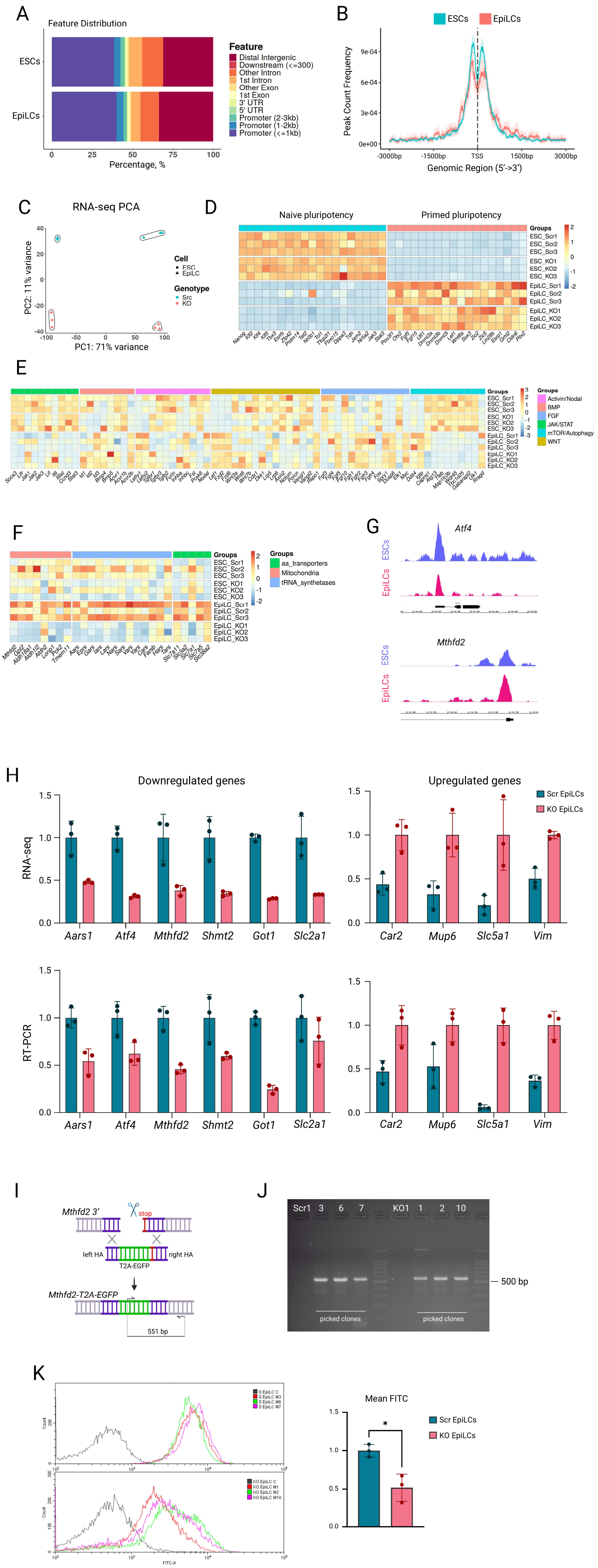

A
Feature Distribution
ESCs
EpiLCs
Feature
Distal Intergenic
Downstream (<=300)
Other Intron
1st Intron
Other Exon
1st Exon
3' UTR
5' UTR
Promoter (2-3kb)
Promoter (1-2kb)
Promoter (<=1kb)
Percentage, %
B
ESCs
EpiLCs
Peak Count Frequency
Genomic Region (5'->3')
C
RNA-seq PCA
PC1: 71% variance
PC2: 11% variance
Cell
ESC
EpiLC
Genotype
Src
KO
D
Naive pluripotency
Primed pluripotency
Groups
E
Groups
Activin/Nodal
BMP
FGF
JAK/STAT
mTOR/Autophagy
WNT
F
Groups
aa_transporters
Mitochondria
tRNA_synthetases
G
Atf4
Mthfd2
ESCs
EpiLCs
H
Downregulated genes
Upregulated genes
Scr EpiLCs
KO EpiLCs
RNA-seq
RT-PCR
Aars1
Atf4
Mthfd2
Shmt2
Got1
Slc2a1
Car2
Mup6
Slc5a1
Vim
I
Mthfd2 3'
stop
left HA
right HA
T2A-EGFP
Mthfd2-T2A-EGFP
551 bp
J
Scr1
3
6
7
KO1
1
2
10
500 bp
picked clones
picked clones
K
Mean FITC
Scr EpiLCs
KO EpiLCs
FITC-A

Figure S3. Omics assay reveals PCBP1 function in amino acid metabolism induction

(A) PCBP1 binding site distribution in ESC and EpiLC genomes revealed by ChIP-seq data analysis. (B) PCBP1 binding site distribution around transcription start sites (TSS). (C) Principal component analysis showing clustering of RNA-seq samples by Scr vs. KO and ESC vs. EpiLC. (D) Heatmap displaying gene expression profiles related to naïve and primed pluripotency in Scr and KO cells (N = 3 biological replicates, i.e. individual cell clones). (E) Heatmap showing expression of genes related to different signaling pathways typical for pluripotent stem cells. (F) Heatmap showing expression of typical targets of the transcription factor ATF4. (G) Integrated genome browser snapshots of ChIP-seq data showing examples of genes occupied by PCBP1 in ESCs and EpiLCs. (H) Validation of the RNA-seq results by RT-PCR. N = 3 biological replicates (individual clones). Error bars indicate SD of the mean. (I) Schematic representation of the *T2A-EGFP* transgene knocked into the *Mthfd2* locus (generated using biorender.com). (J) Genotyping of picked clones for the *T2A-EGFP* insertion. Parental Scr1 and KO1 clones were used as controls. (K) Left panel: flow cytometry analysis comparing EGFP signal among the *Mthfd2-T2A-EGFP* clones in the EpiLC state. Right panel: quantification of EGFP signal presented in the left panel. N = 3 biological replicates (individual cell clones). Error bars indicate SD of the mean. * $p < 0.05$.

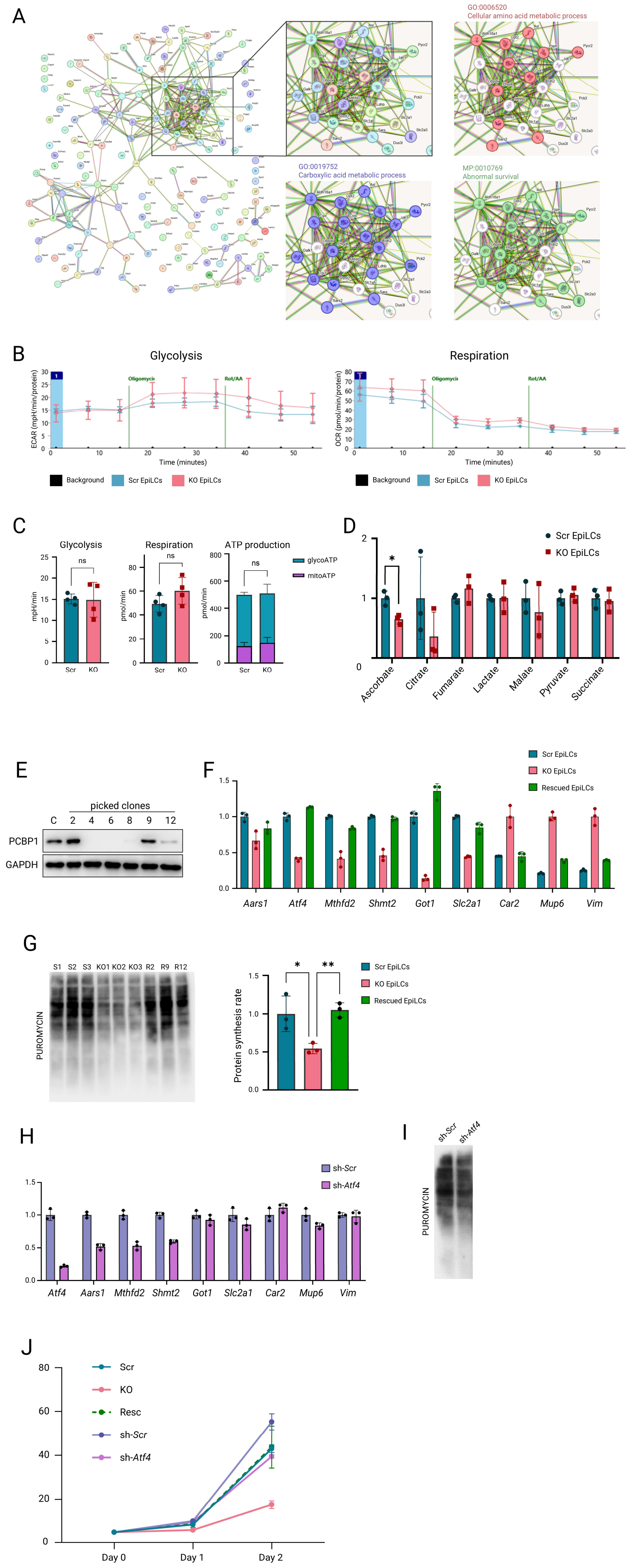
A
GO:0006520
Cellular amino acid metabolic process
GO:0019752
Carboxylic acid metabolic process
MP:0010769
Abnormal survival
B
Glycolysis
Respiration
ECAR (mpH/min/protein)
OCR (pmol/min/protein)
Oligomycin
Rot/AA
Time (minutes)
Background
Scr EpiLCs
KO EpiLCs
C
Glycolysis
Respiration
ATP production
ns
mpH/min
pmol/min
glycoATP
mitoATP
Scr
KO
D
Scr EpiLCs
KO EpiLCs
Ascorbate
Citrate
Fumarate
Lactate
Malate
Pyruvate
Succinate
E
picked clones
C 2 4 6 8 9 12
PCBP1
GAPDH
F
Scr EpiLCs
KO EpiLCs
Rescued EpiLCs
Aars1
Atf4
Mthfd2
Shmt2
Got1
Slc2a1
Car2
Mup6
Vim
G
S1 S2 S3 KO1 KO2 KO3 R2 R9 R12
PUROMYCIN
Protein synthesis rate
Scr EpiLCs
KO EpiLCs
Rescued EpiLCs
H
sh-Scr
sh-Atf4
Atf4
Aars1
Mthfd2
Shmt2
Got1
Slc2a1
Car2
Mup6
Vim
I
sh-Scr
sh-Atf4
PUROMYCIN
J
Scr
KO
Resc
sh-Scr
sh-Atf4
Day 0
Day 1
Day 2

Figure S4. PCBP1 is crucial for protein synthesis in priming pluripotent cells

(A) Clustering of differentially abundant proteins in EpiLCs using STRING (string-db.org), with several enriched pathways highlighted. (B) ECAR (glycolysis) and OCR (respiration) measurements using the SeaHorse XFe96 analyzer. (C) Energy metabolism of Scr and KO EpiLCs using a SeaHorse analyzer, measuring glycolysis (ECAR), respiration (OCR), and calculated ATP production. N = 4 technical replicates. Error bars indicate SD of the mean. (D) Metabolome analysis of carboxylic acids in Scr and KO EpiLCs. N = 3 biological replicates (individual clones). Error bars indicate SD of the mean. $^*p<0.05$. (E) Western blot analysis of the picked clones for PCBP1 after knocking exogeneous *Pcbp1* into the *Rosa26* locus. (F) Representative results of RT-PCR analysis aimed at verification of restoration of gene expression in *Pcbp1*-rescued EpiLCs. n = 3 technical replicates. Error bars indicate SD of the mean. (G) Left panel: SUnSET assay showing restoration of the protein synthesis rates in *Pcbp1*-rescued EpiLCs. Right panel: quantification analysis of Puromycin signal presented on the left panel. N = 3 biological replicates (individual clones). Error bars indicate SD of the mean.; $^*p<0.05$; $^{**}p<0.01$. (H) RT-PCR analysis of control EpiLCs (shScr) and with shRNA-mediated *Atf4* knockdown (shAtf4). n = 3 technical replicates. Error bars indicate SD of the mean. (I) SUnSET assay showing protein synthesis rate in control EpiLCs (sh-*Scr*) and with *Atf4* knockdown (sh-*Atf4*). (J) Proliferation rates of Scr, KO, Rescued, sh-*Scr*, and sh-*Atf4* cells during the naïve-to-primed pluripotency transition. For Scr, KO, and Resc N = 3 biological replicates (individual clones); for sh-*Scr* and sh-*Atf4* n=3 (technical replicates); error bars indicate SD of the mean.

Supplemental Tables

Table S1. Oligonucleotides used for *Pcbp1* knockdown and analyses in pre-implantation embryos

Control siRNA	GCACCCGATAAGCGGTCAA
Pcbp1 siRNA-1	GTGTGACTGAAAGCGGACTCA
Pcbp1 siRNA-3	GAACCAGGTGGCAAGACAA
Pcbp1 siRNA-5	GCTGATGCACGGAAAGGAA
Hprt1	Mm00446968_m1
Pou5f1	Mm00658129_gH
Pcbp1	Mm00478712_s1
5' *GFP*	CCACATGAAGCAGCACGACT
3' *GFP*	TGCGCTCCTGGACGTAGC
GFP Probe	6-FAM-TTCAAGTCCGCCATGCCCGAA-TAMRA

Table S2. Oligonucleotides used for generation and analysis of *Pcbp1*-heterozygous mice

gRNA#1 (nickase)	gTCGGCTGCTGATGCACGGAA (AGG)
gRNA#2 (nickase)	gACACCGGCGTCCATGGCGAG (CGG)
ssODN-1 (nickase)	CCTCGCGGATCCTCTTCACCGACTCCCCTTTCTTCCCGATGAT<br>GCTGCCTACTTCCTTTCGAGCGGCGGGCGGCGTTCGGGAGTT<br>GGGCTCGTTACGTGGTCAAGTCCTGGGCGGCTGGC
gRNA#3 (nuclease)	gTCAACGTGACTCTCACCATT (CGG)
ssODN-2 (nuclease)	ATGATGCTGCCTACTTCCTTTCCGTGCATCAGCAG**g**CGAATG<br>GTGAGAGTCACGTTGAGTCCGCTTTCAGT
Pcbp1-gen_F	ACTTGACCACGTAACGAGCC
Pcbp1-gen_R	CCCCTCCGAGATGTTGATCC

Table S3. CRISPR/Cas9 and knockdown in ESCs

sh-*Scr* (Addgene #1864)	CCTAAGGTTAAGTCGCCCTCGCTCGAGCGAGGGC GACTTAACCTTAGG
sh-*Atf4* (Zhao et al., 2025)	CCGGCCATCTCCCAGAAAGTTTAATCTCGAGATTA AACTTTCTGGGAGATGGTTTTTG
CRISPR-gRNA_*Mthfd2*-KI_F	caccgCTCAGAGTGCTGCTAGTTGG
CRISPR-gRNA_*Mthfd2*-KI_R	aaacCCAACTAGCAGCACTCTGAGc

Table S4. Oligonucleotides used in RT-PCR

Atf4_F	GATGGGTTCTCCAGCGACAAG
Atf4_R	CCGGAAAAGGCATCCTCCTT
Mthfd2_F	TCCTTGTTGTCTGCGTTGGC
Mthfd2_R	CTTCATTTCGCACTGCCGCC
Aars1_F	TTGCTATTCCCTCGGAGCAC
Aars1_R	CTCCTCGGGAACCTTAGCTC
Got1_F	AAGCAGATCGCTGCTGTCAT
Got1_R	AATAGCGAATAGCCCACGCA
Shmt2_F	AGTACGAACCGTAGACCCCA
Shmt2_R	GTACTCGCGGAACATAGGGG
Slc2a1_F	ACCTCTTCCGAACCGACAGAT
Slc2a1_R	TCTGGAGCCATCAAAGTCCTG
Vim_F	CGGCTGCGAGAGAAATTGC
Vim_R	CCACTTTCCGTTCAAGGTCAAG
Car2_F	CTCTGCTGGAATGTGTGACCTG
Car2_R	CCAGTTGTCCACCATCGCTTCT
Mup6_F	GGAAACCTTCCAGCTGATGTCG
Mup6_R	CTCTAATGATTCCATGCTCCTCAC
Gapdh_F	AGGTCGGTGTGAACGGATTTG
Gapdh_R	TGTAGACCATGTAGTTGAGGTCA

Zhao, L., Chen, Y., Ding, X., Li, H., and Li, J. (2025). Targeting Atf4 for enhanced neuroprotection: Role of quercetin-loaded EVs in ischemic stroke. J Pharm Anal *15*, 101312. 10.1016/j.jpha.2025.101312.
