# Supplementary material for: PCBP1 orchestrates amino acid metabolism burst during the naïve-to-primed pluripotency transition
Source: Stem Cell Reports. 2026 Jun 4;21(7):102950. doi: 10.1016/j.stemcr.2026.102950 (PMC13385435; doi:10.1016/j.stemcr.2026.102950)
Supplement: Document S2. Article plus supplemental information [file mmc3.pdf]

STEM CELL REPORTS
Article
OPEN ACCESS

PCBP1 orchestrates amino acid metabolism burst during the naïve-to-primed pluripotency transition

Evgeny I. Bakhmet,[1,17,*] Evgeniy V. Potapenko,[2,12] Oleg Y. Shuvalov,[3] Arseniy A. Lobov,[4,13] Egor A. Repkin,[5] Daria V. Kriger,[6] Nadezhda E. Vorobyeva,[7] Alexey N. Korablev,[8,14] Anna S. Zinovyeva,[1] Andrey A. Kuzmin,[2] Nikolay D. Aksenov,[9] Arthur T. Kopylov,[10] Guangming Wu,[11,15,16] Hans R. Schöler,[11] and Alexey N. Tomilin[2,*]

[1]Pluripotency Dynamics Group, Laboratory of the Molecular Biology of Stem Cells, Institute of Cytology, Russian Academy of Sciences, St-Petersburg 194064, Russia
[2]Laboratory of the Molecular Biology of Stem Cells, Institute of Cytology, Russian Academy of Sciences, St-Petersburg 194064, Russia
[3]Oncopharmacology Group, Laboratory of Regulation of Gene Expression, Institute of Cytology, Russian Academy of Sciences, St-Petersburg 194064, Russia
[4]Laboratory of Regenerative Biomedicine, Institute of Cytology, Russian Academy of Sciences, St-Petersburg 194064, Russia
[5]Resource Centre for Molecular and Cell Technologies, Research Park, St-Petersburg State University, St-Petersburg 199034, Russia
[6]Regulated Proteolysis Group, Laboratory of Cell Metabolism and Signaling, Institute of Cytology, Russian Academy of Sciences, St-Petersburg 194064, Russia
[7]Group of Transcriptional Complexes Dynamics, Institute of Gene Biology, Russian Academy of Sciences, Moscow 119334, Russia
[8]Department of Molecular Mechanisms of Development, Institute of Cytology and Genetics, Russian Academy of Sciences, Siberian Branch, Novosibirsk 630090, Russia
[9]Department of Intracellular Signaling and Transport, Institute of Cytology, Russian Academy of Sciences, St-Petersburg 194064, Russia
[10]Laboratory of Structural Proteomics, V.N. Orekhovich Institute of Biomedical Chemistry, Russian Academy of Sciences, Moscow 119121, Russia
[11]Department of Cell and Developmental Biology, Max Planck Institute for Molecular Biomedicine, Münster 48149, Germany
[12]Present address: Department of Evolutionary and Environmental Biology, University of Haifa, Haifa, 3498838, Israel
[13]Present address: Department of Systems Immunology, Weizmann Institute of Science, Rehovot, 7610001, Israel
[14]Present address: State Research Center of Virology and Biotechnology "Vector", Federal Service for Surveillance on Consumer Rights Protection and Human Well-Being, Koltsovo, Moscow 630559, Russia
[15]Present address: The Third Affiliated Hospital of Guangzhou Medical University, Guangzhou, Guangdong 510150, China
[16]Present address: Division of Basic Research, Guangzhou National Laboratory, Guangzhou 510005, China
[17]Lead contact
*Correspondence: e.bakhmet@incras.ru (E.I.B.), a.tomilin@incras.ru (A.N.T.)
https://doi.org/10.1016/j.stemcr.2026.102950

SUMMARY

Embryo implantation is accompanied by the naïve-to-primed pluripotency state transition in epiblast cells—the process involving proliferation and anabolic boost, needed for extensive embryo growth. Here, we show that *Pcbp1* knockout leads to embryo growth arrest shortly after implantation. By modeling the naïve-to-primed pluripotency transition *in vitro*, we observe impaired proliferation and induction of apoptosis in cells deficient for PCBP1. Using multi-omics approaches, we reveal a crucial role for PCBP1 in driving a transcriptional burst of numerous genes involved in the import and the *de novo* synthesis of essential and conditionally essential amino acids. PCBP1 deficiency is consequently associated with a slowdown in protein biosynthesis, which explains the early lethality of the knockout embryos. Our findings thus uncover the molecular mechanisms underlying anabolic changes during the naïve-to-primed pluripotency transition and highlight the essential role of PCBP1 in this critical process.

INTRODUCTION

Early mammalian embryogenesis is characterized by a prolonged period without significant body growth. In mice, prenatal development lasts approximately 20 days, with the first five days after fertilization (E5) consisting solely of successive cleavage divisions. Implantation then establishes the embryo-uterine interaction, providing all essential nutrients for rapid embryo growth. During implantation, pluripotent stem cells of the epiblast undergo the naïve-to-primed pluripotency transition (Brons et al., 2007; Han et al., 2010; Hayashi et al., 2011; Tesar et al., 2007), a process characterized by changes in the epigenomic (Lando et al., 2024), gene expression (Boroviak et al., 2014; Santini et al., 2024), and signaling pathways (Huang et al., 2017; Zhao et al., 2024). At this time, epiblast cells become receptive to external differentiation signals (Du and Wu, 2024; Pera and Rossant, 2021; Weinberger et al., 2016), preparing for gastrulation. While energy metabolism alterations such as enhanced aerobic glycolysis were shown during this phase (Luo et al., 2023a; Mathieu and Ruohola-Baker, 2017; Zhou et al., 2012), there is still no data describing anabolic changes facilitating rapid embryo growth after implantation.

PCBP1 is a member of the KH-domain poly(C)-DNA/RNA-binding protein family. This is a ubiquitously expressed protein with a variety of functions including transcriptional regulation (Karam et al., 2024; Mohanty et al., 2021), mRNA stability (Ishii et al., 2020; Van Nostrand et al., 2020), alternative splicing (Huang et al., 2021; Krapacher et al., 2022; Tripathi et al., 2016; Wang et al., 2024), translation (Chaudhury et al., 2010; Hussey

© 2026 The Authors.
Published by Elsevier Inc. on behalf of International Society for Stem Cell Research.
This is an open access article under the CC BY license (http://creativecommons.org/licenses/by/4.0/).

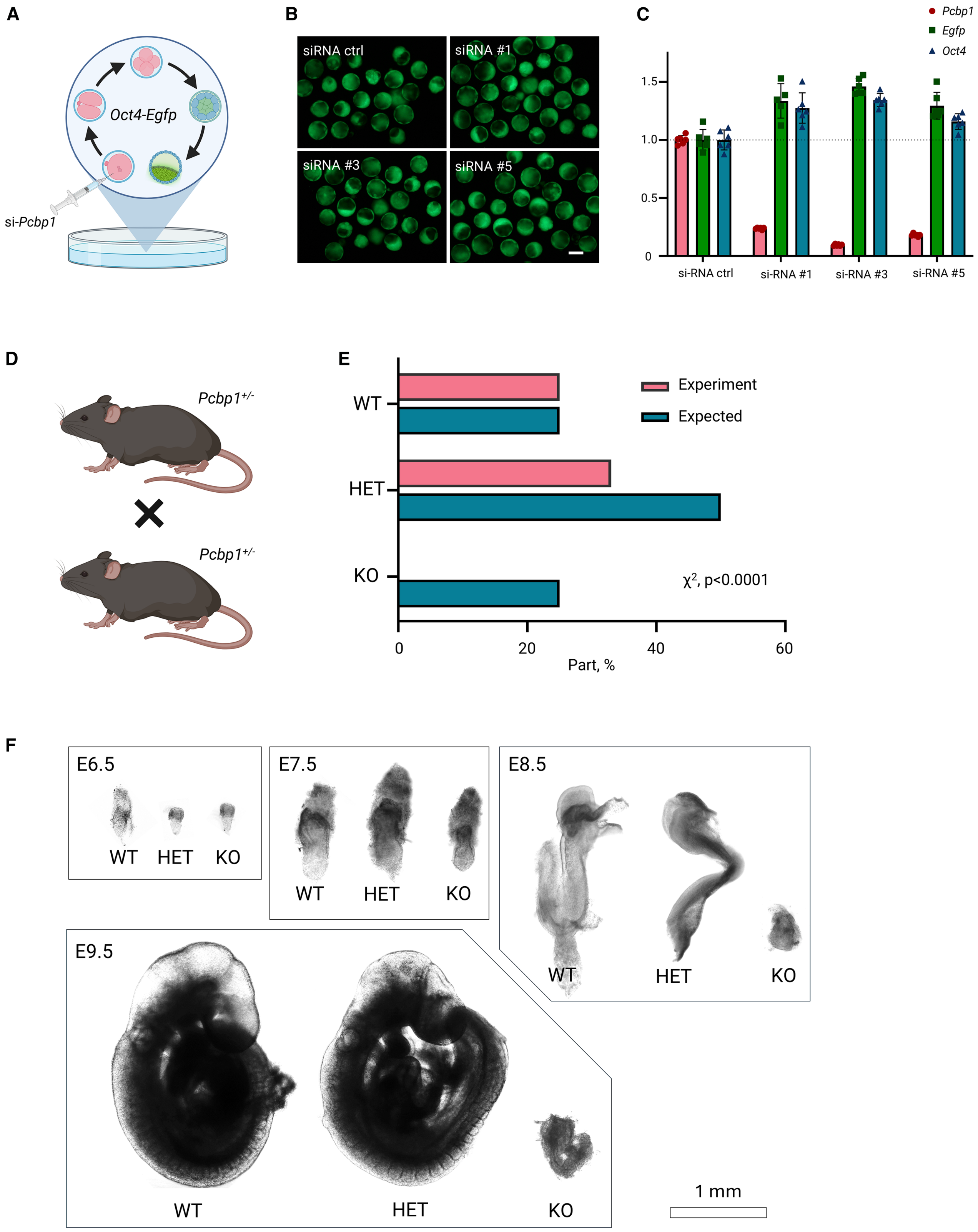

(legend on next page)

et al., 2011), and iron metabolism (Bae et al., 2020; Patel et al., 2019; Wang et al., 2023). Several studies indicate its role in erythropoiesis and tumorigenesis, i.e., in highly proliferative cells (Ji et al., 2021; Lee et al., 2022; Tripathi et al., 2016). Previously, we demonstrated that another KH-domain protein, HNRNP-K, targets open chromatin in mouse naive embryonic stem cells (ESCs) corresponding to epiblast before implantation but is not required for pluripotency maintenance; rather, it plays a broader role in ESC viability (Bakhmet et al., 2019). In turn, ESCs with *Pcbp1* knockout (KO) are viable (Bakhmet et al., 2020), though *Pcbp1*-deficient embryos are not observed for a few days after implantation (Ghanem et al., 2015). This raises the question of whether PCBP1 has specific functions during the naïve-to-primed pluripotency transition of epiblast cells. Here, we show that growth arrest of PCBP1-deficient embryos is observed after implantation, while *in vitro* priming of the KO ESCs results in a failed proliferation boost, as well as in cell death by apoptosis. Multi-omics assays unveil a crucial role of PCBP1 in the intensification of import and *de novo* synthesis of essential and conditionally essential amino acids during the naïve-to-primed pluripotency transition of epiblast, required for protein biosynthesis and therefore, intensive embryonic growth after implantation.

RESULTS

Pcbp1 loss leads to embryonic growth arrest after implantation

To investigate the role of PCBP1 in early development, we first sought to determine why *Pcbp1* KO embryos exhibited peri-implantation lethality (Ghanem et al., 2015). To rule out the possibility that maternal *Pcbp1* mRNA rescues pre-implantation development, we knocked it down (along with its embryonic counterpart) by injecting *Pcbp1* siRNAs into oocytes (Figure 1A) of the OG2 mice harboring an *Oct4-EGFP* transgene (Szabó et al., 2002). After culturing, we observed that embryos injected with *Pcbp1* siRNA developed into blastocysts at a similar rate as those injected with control siRNA (Figure 1B). RT-PCR analysis confirmed efficient *Pcbp1* mRNA depletion (Figure 1C). Notably, both *Oct4* and *EGFP* mRNA expression are properly initiated in *Pcbp1*-knockdown blastocysts, even though PCBP1 is known to occupy *Oct4* regulatory elements (Bakhmet et al., 2017). In sum, these findings indicate that PCBP1 is dispensable for both *Oct4* expression onset and pre-implantation development.

Next, we used CRISPR/Cas9-mediated gene targeting to generate *Pcbp1*-heterozygous (HET) mice carrying indels at the beginning of the protein-coding sequence (Figures 1D, S1A, and S1B). Consistent with previous findings (Ghanem et al., 2015), genotyping of 2-week-old pups from HET intercrosses revealed no KO offspring and a reduced frequency of HET mice compared to expected Mendelian ratios (Figure 1E). To further examine the effects of *Pcbp1* knockout, we performed morphological analysis of post-implantation (E6.5-E9.5) embryos from HET intercrosses (Figures 1F and S1C). While WT and HET embryos exhibited a progressive size increase, KO embryos demonstrated complete growth arrest from E6.5 onward with the absence of morphologically distinct features.

These findings demonstrate that *Pcbp1* gene function loss does not affect pre-implantation development. However, PCBP1-deficient embryos exhibit an early lethal phenotype, likely due to severe growth defects following implantation.

Pcbp1 knockout disrupts the naïve-to-primed pluripotency transition in ESCs

We hypothesized that the observed *Pcbp1* KO phenotype results from defects in the naïve-to-primed pluripotency transition occurring at and shortly after implantation. To investigate this, we utilized a well-established model recapitulating this transition *in vitro* and relying on the conversion of ESCs into epiblast stem cells (EpiSCs) via the intermediate epiblast-like stem cell (EpiLC) stage (Figure 2A) (Guo et al., 2009; Tosolini and Jouneau, 2016). To this end, we used KO *Pcbp1* and control (Scr) ESCs, previously generated using CRISPR/Cas9 (Bakhmet et al., 2020). No

Figure 1. Loss of Pcbp1 function leads to embryo lethality due to impaired post-implantation growth

(A) Schematic representation of the experimental design for *Pcbp1* mRNA knockdown in pre-implantation OG2 (*Oct4-GFP*) embryos. The figure is generated using biorender.com.

(B) Fluorescent imaging of OG2 blastocysts derived from zygotes injected with either *Pcbp1* mRNA-targeting siRNAs (#1, 3, 5) or control siRNA (ctrl). Scale bar, 100 μm.

(C) TaqMan PCR analysis of the blastocysts shown in (B), displaying normalized expression levels of the indicated mRNAs. Data represents $N = 2$ biological and $n = 3$ technical (for each N) replicates; error bars indicate SD of the mean.

(D) Schematic representation of *Pcbp1* heterozygous mice crossbreeding (generated using BioRender.com).

(E) Observed and expected percentages of wild-type (WT), heterozygous (HET), and homozygous (KO) *Pcbp1* pups at 2 weeks of age following intercrossing of HET mice. A chi-square statistical test revealed a significant difference between expected and observed ratios; $N = 115$; $p < 0.0001$.

(F) Microphotographs demonstrate a striking difference in embryo size between KO and WT/HET embryos from E6.5 onward.

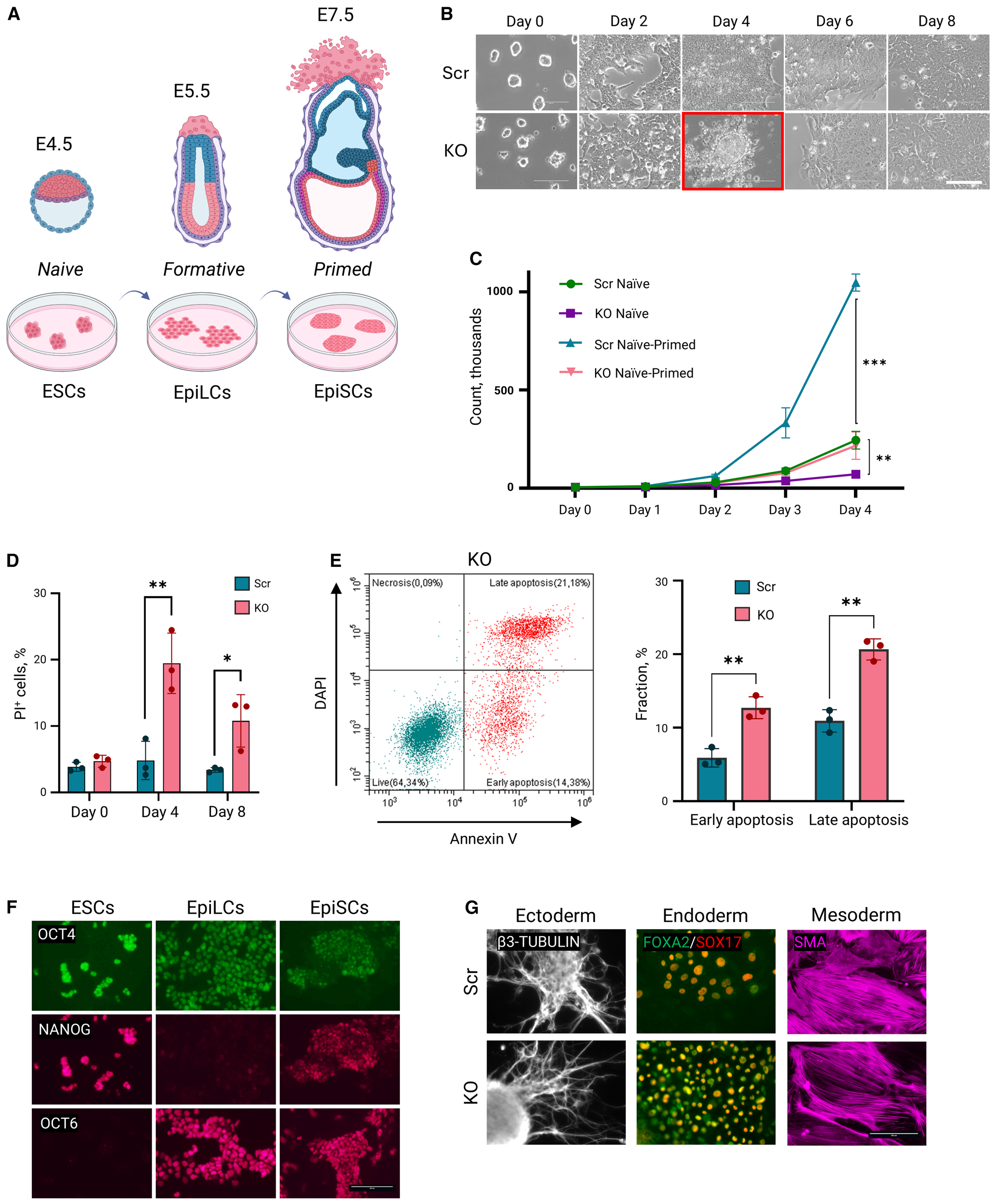

(legend on next page)

visible differences between Scr and KO cells were observed until Day 2, corresponding to the formative EpiLC stage. However, by Day 4, KO cells exhibited increased cell death and aggregation (Figure 2B). Time-lapse microscopy of Scr cells seeded at low density and cultured under priming conditions showed monolayer formation by Day 4, whereas KO cells demonstrated near-complete proliferation arrest at this stage, followed by partial proliferation recovery (Video S1). Proliferation analysis confirmed that, compared to Scr ESCs, KO cells exhibited a reduced growth rate even in the naive state (Figure 2C). This difference became significantly more pronounced during the naïve-to-primed transition: during this period, Scr cells underwent a proliferation burst while KO cells only reached proliferation rates typical of Scr ESCs (Figure 2C). Surprisingly, there were no differences between Scr and KO cells in G0/G1, S, and G2/M cell cycle phase distribution on Days 0 and 2, suggesting a general cell growth deceleration rather than cell-cycle arrest (Figure S2A). However, propidium iodide staining confirmed an increase in KO cell death on Day 4, reaching 20% of the population (Figure 2D), while ANNEXIN V staining revealed that these cells underwent apoptosis (Figure 2E).

Despite the aforementioned perturbations, KO cells in different pluripotency states showed normal expression of the corresponding markers: ESCs expressed OCT4 and NANOG, EpiLCs expressed OCT4 and OCT6, and EpiSCs were positive for all three markers (Figure 2F). Western blot analysis of ESC and EpiLC lysates confirmed these results and also showed a significant upregulation of PCBP1 in Scr cells in the formative pluripotency state (Figure S2B). Notably, KO cells could also be maintained in the formative state using A_{lo}XR medium (Kinoshita et al., 2020). The initial days of culture were accompanied by impaired propagation and visible cell death (Figure S2C). Nevertheless, KO cells could be successfully maintained under these conditions for up to one month, although only seven passages were achieved, compared to sixteen passages for Scr cells over the same period (Figure S2C). Immunocytochemistry analysis confirmed OCT4 and OCT6 expression in KO formative cells, while NANOG was expectedly downregulated in these cells (Figure S2D). Consistent with our previous teratoma analysis (Bakhmet et al., 2020), KO cells retained the ability to give rise to derivatives of ectoderm, endoderm, and mesoderm *in vitro* (Figure 2G).

In sum, our findings indicate that PCBP1 is essential for sustaining viability and proliferation during the transition between the pluripotency substates. The data presented below argue that this is due to failed adaptation to changing metabolic requirements.

PCBP1 drives the expression burst of genes involved in amino acid and carbohydrate metabolism during the priming of pluripotent stem cells

To uncover the molecular mechanisms affected by PCBP1 deficiency during the naïve-to-primed pluripotency transition, we applied a multi-omics approach at early stages before visible morphological defects emerged, specifically, on Day 0 (ESCs) and Day 2 (EpiLCs) (Figure 3A). Considering that PCBP1 is a DNA-binding transcription factor, we first assessed PCBP1 genome occupancy changes during this transition using ChIP-seq analysis. *De novo* motif search revealed an expected enrichment for poly-C sites in various compositions (Figure 3B). Previously, we demonstrated that another KH-domain protein, HNRNP-K, binds to open, transcriptionally active chromatin in ESCs (Bakhmet et al., 2019). Similarly, PCBP1 co-localized to open chromatin (ATAC-seq), active enhancers, and transcriptionally active genes (H3K27ac and H3K4me3) in both ESCs and EpiLCs but was absent from heterochromatin regions marked by H3K9me3 and H3K27me3 modifications (Figure 3C). The vast majority of PCBP1 binding sites, both in ESCs and

Figure 2. PCBP1 is essential for the proliferation burst and cell viability during the naïve-to-primed pluripotency transition
(A) Illustration of murine embryos at peri-implantation stages aligned with corresponding stages of cultured pluripotent stem cells (generated using biorender.com).
(B) Microphotographs of Scr and KO cells undergoing the naïve-to-primed pluripotency transition. The red rectangle highlights aggregated and dying KO cells on Day 4, a defining feature of these cells. Scale bars, 100 μm.
(C) Proliferation rates of Scr and KO cells in naive culture conditions and during the naïve-to-primed pluripotency transition. $N = 3$, biological replicates (individual clones); error bars indicate SD of the mean; $^{**}p < 0.01$ and $^{***}p < 0.001$.
(D) Propidium iodide (PI) staining to identify dead cells in the naive state (Day 0) and during the naïve-to-primed pluripotency transition (Days 4 and 8). $N = 3$, biological replicates (individual cell clones); error bars indicate SD of the mean; $^{*}p < 0.05$ and $^{**}p < 0.01$.
(E) Left: flow cytometry analysis of the KO cells on Day 4 of the naïve-to-primed pluripotency transition stained with DAPI and antibodies against ANNEXIN V to identify apoptotic cells. Right: quantification analysis of cells positive either for ANNEXIN V only (early apoptosis) or for Annexin V and DAPI (late apoptosis). $N = 3$, biological replicates (individual cell clones). Error bars indicate SD of the mean; $^{**}p < 0.01$.
(F) Immunofluorescence microscopy of *Pcbp1* KO ESCs, EpiLCs, and EpiSCs stained with OCT4, NANOG, and OCT6 antibodies. Scale bars, 100 μm.
(G) *In vitro*-differentiated Scr and KO cells stained for markers of ectoderm (β3-TUBULIN —neurons), endoderm (FOXA2/SOX17—definitive endoderm), and mesoderm (αSMA—smooth muscle cells). Scale bars, 100 μm.

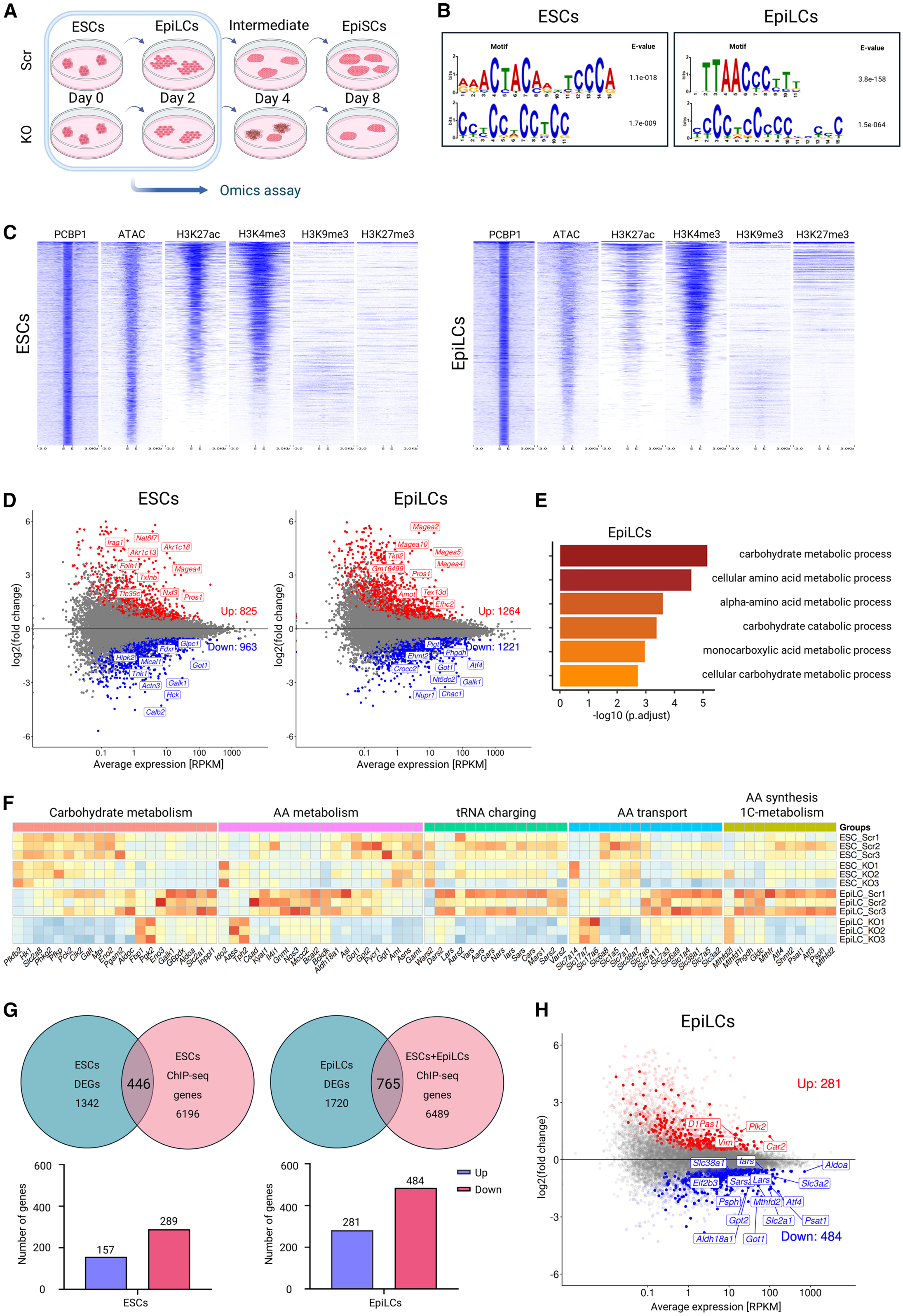

(legend on next page)

EpiLCs, were found within promoters (<1 kb) (Figure S3A) around transcription start sites (TSSs) (Figure S3B).

Next, we performed RNA-seq analysis on Scr and KO cells at the ESC and EpiLC stages using three independent Scr and KO clones. Principal component analysis (PCA) demonstrated clear clustering of samples along two primary components—ESCs vs. EpiLCs and Scr vs. KO (Figure S3C). Differential expression analysis revealed 963 downregulated and 825 upregulated genes in ESCs and 1,221 downregulated and 1,264 upregulated genes in EpiLCs (Figure 3D). Key metabolic genes were significantly downregulated, including *Atf4*, *Got1*, and *Phgdh*, which play crucial roles in amino acid metabolism, and *Galk1*, which is involved in galactose catabolism (Figure 3D). Consistent with the above findings (Figures 2F and S2B), no changes in expression were detected for mRNAs associated with naive pluripotency (*Klf4*, *Nanog*, *Esrrb*, *Zfp42*, *Tbx3*) or primed pluripotency (*Fgf5*, *Otx2*, *Pou3f1*, *Dnmt3a*, *Dnmt3b*), suggesting that the developmental transition proceeded successfully (Figure S3D). Additionally, we did not detect any notable differences in the expression of genes involved in signaling pathways characteristic of pluripotent stem cells (Figure S3E). Gene ontology (GO) analysis revealed that several metabolic pathways were affected in KO EpiLCs (Figure 3E). The two most prominently disrupted pathways upon *Pcbp1* gene function loss were related to amino acid and carbohydrate metabolism (Figure 3E). Associated with these pathways mRNAs include those related to glycolysis/gluconeogenesis (*Eno2*, *Eno3*, *Pfkp*, *Aldoa*, *Aldoc*, *Slc2a1*, *Galk1*, *Galt*), amino acid metabolism (*Got1*, *Gpt2*, *Aldh18a1*, *Nos3*, *Pycr1*), amino acid transport (*Slc1a4*, *Slc3a2*, *Slc7a3*, *Slc7a5*, *Slc7a6*), serine/glycine *de novo* synthesis and folate metabolism (*Atf4*, *Phgdh*, *Psph*, *Shmt2*, *Mthfd2*, *Psat1*, *Gldc*), as well as tRNA charging (*Iars*, *Sars*, *Sars2*, *Lars*, *Vars2*) (Figure 3F). Interestingly, while Scr EpiLCs mostly exhibited significant upregulation of these mRNAs, KO EpiLCs showed no expression change or slight downregulation (Figure 3F). As expected, the reduced expression of *Atf4* in KO EpiLCs (compared to Scr EpiLCs) correlated with the reduced expression of known ATF4 target genes, including several regulators of one-carbon (1C) metabolism, amino acid transport, and tRNA charging (Figure S3F).

Next, we investigated genes whose transcription may be directly regulated by PCBP1. We focused on differentially expressed genes (DEGs) bound by PCBP1 according to our ChIP-seq data. For this, we cross-referenced ESC DEGs with ESC genes exhibiting PCBP1 binding within a 10-kb distance from the TSS, while for EpiLC DEGs, we focused on genes occupied by PCBP1 in both ESCs and EpiLCs (Figure 3G). We hypothesized that PCBP1 binding in ESCs could influence gene expression in EpiLCs. The analysis identified 446 potential direct PCBP1 targets in ESCs and 765 potential direct PCBP1 targets in EpiLCs. Notably, most of these genes were downregulated in KO cells, suggesting that PCBP1 functions primarily as a transcriptional activator rather than a repressor (Figure 3G, lower panel). Among the most notable potential PCBP1 targets were genes related to amino acid and carbohydrate metabolism, including *Atf4*, *Aldh18a1*, *Slc38a1*, *Got1*, *Sars2*, *Lars*, *Mthfd2*, *Psat1*, *Aldoa*, and *Slc2a1* (Figures 3H and S3G). Expression changes in several of these genes were further validated by RT-PCR, confirming both downregulation and upregulation patterns (Figure S3H). In addition, using CRISPR/Cas9, we generated three *Mthfd2-T2A-EGFP* clones of both Scr and KO cells (Figures S3I and S3J) and confirmed a reduced EGFP signal in KO EpiLCs by flow cytometry (Figure S3K).

Taken together, these results indicate that while KO cells successfully undergo the naïve-to-primed developmental transition, they fail to achieve the necessary amino acid and carbohydrate metabolism intensification during this process. Given the observed genome-wide

Figure 3. PCBP1 regulates the expression burst of genes related to amino acid and carbohydrate metabolism in pluripotent stem cells undergoing the naïve-to-primed transition

(A) Schematic representation of timepoints used in omics analyses to identify the molecular mechanisms affected in KO cells. Generated using biorender.com.

(B) PCBP1 *de novo* motifs identified from the ChIP-seq data using the MEME suite at the ESC and EpiLC stages.

(C) Heatmaps show PCBP1 binding signals in open and transcriptionally active chromatin. ATAC-seq and ChIP-seq data for histone marks in ESCs and EpiLCs were included in the analysis. “S” indicates peak start; “E” indicates peak end.

(D) Expression plots of KO differentially expressed genes at ESC and EpiLC stages. The names of the top 20 most significantly differentially expressed genes are highlighted.

(E) Gene ontology analysis reveals the top 6 pathways affected in KO cells at the EpiLC stage.

(F) Heatmaps show differentially expressed genes (DEGs) associated with the most affected metabolic processes. $N = 3$, biological replicates (individual cell clones).

(G) Intersection of genes occupied by PCBP1 in ChIP-seq (±10 kb from TSS) with those differentially expressed in KO cells. ESC DEGs were compared with the ESC ChIP-seq dataset, whereas EpiLC DEGs were compared with both ESC and EpiLC ChIP-seq datasets.

(H) Expression plot represents differentially expressed genes with a PCBP1 physically binding nearby (±10 kb, highlighted dots). Semi-transparent dots represent non-occupied differentially expressed genes.

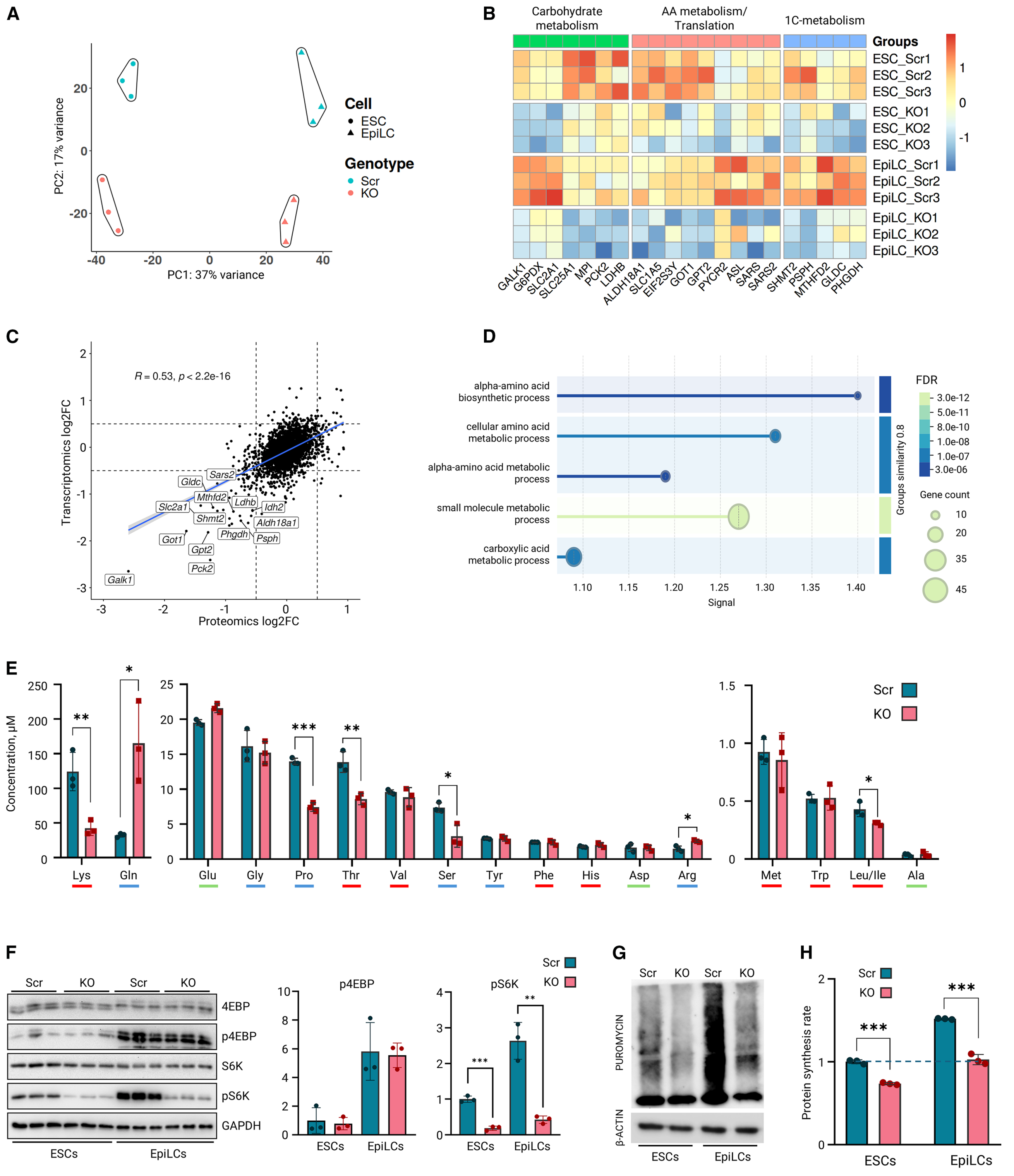

Figure 4. *Pcbp1* loss disrupts amino acid metabolism, leading to a decline in protein synthesis during the naive-to-primed pluripotency transition

(A) Principal component analysis shows clustering of samples based on protein abundance comparing Scr vs. KO and ESC vs. EpiLC principal components.

(legend continued on next page)

occupation of PCBP1, we propose that this metabolic activation is primarily driven by the PCBP1-mediated transcriptional activation of key amino acid and carbohydrate metabolism genes.

Pcbp1 loss impairs protein biosynthesis during pluripotent stem cell priming

To validate the above findings and assess the physiological state of PCBP1-deficient cells, we conducted several tests. First, we used a shotgun proteomics approach to analyze protein abundance in Scr and KO cells. As observed in the RNA-seq data, PCA revealed a clear separation between ESCs and EpiLCs, as well as between Scr and KO samples (Figure 4A). Consistent with the RNA-seq data, Scr EpiLCs (compared to Scr ESCs) showed an upregulation of several key metabolic proteins, including PYCR2, SARS2, MTHFD2, PHGDH, related to amino acid metabolism, as well as GALK1 and SLC2A1, related to carbohydrate metabolism (Figure 4B). In contrast, KO EpiLCs exhibited minimal changes or even downregulation of these proteins. To further correlate RNA-seq and proteomics data, we compared the Log2-fold change (FC) of mRNA and protein expression in EpiLCs (Figure 4C). Most mRNAs and their corresponding proteins identified by both approaches showed similar Log2-FC values and correlated positively with each other (Figure 4C). As the most notable changes were observed for EpiLCs, we focused further analysis on these cells. GO analysis of differentially expressed proteins highlighted amino acid metabolism as one of the most affected pathways (Figure 4D). Additionally, a protein-protein interaction network reconstruction via the String database (Szklarczyk et al., 2021) revealed that the central cluster of differentially abundant proteins was enriched for proteins associated with amino acid and carboxylic acid metabolism, with most linked to abnormal survival phenotypes (Figure S4A).

Given the downregulation of genes and proteins involved in carbohydrate and carboxylic acid metabolism, along with the observed proliferation decline in KO cells (Figure 2C), we hypothesized a possible energy deficiency in these cells. To test this, we measured glycolysis (ECAR) and oxygen consumption rate (OCR, a measure of respiration) using a SeaHorse analyzer in Scr and KO EpiLCs (Figures S4B and S4C). However, we observed no significant differences in glycolysis, respiration, or calculated ATP production. One possible explanation is that KO cells exhibit reduced proliferation due to limited nutrient supply, while engaging compensatory mechanisms that adjust energy metabolism accordingly.

Given the potential disruption in amino acid and carboxylic acid levels in PCBP1-deficient cells, we performed metabolome analysis. The results showed reduced levels of lysine, proline, serine, leucine/isoleucine, and threonine, along with increased levels of glutamine and arginine (Figure 4E). Notably, all these amino acids are either essential or conditionally essential. Consistent with the results of energy metabolism (Figures S4B and S4C), no significant changes were detected in the levels of citrate, succinate, fumarate, malate, lactate, and pyruvate (Figure S4D). Rapid proliferation and anabolic activity are typically associated with the activation of mTOR signaling, which also functions as a sensor of amino acid availability. We therefore examined the phosphorylation status of its key effectors, 4EBP and S6K. As expected, both p4EBP and pS6K were strongly elevated in control EpiLCs, indicating robust mTOR activation (Figure 4F). In contrast, although p4EBP was similarly upregulated in KO EpiLCs, pS6K levels were markedly reduced compared to the Scr control, suggesting impaired mTOR signaling in these cells. Together, the observed imbalance in amino acid levels and reduced mTOR activity point to decreased protein biosynthesis in KO EpiLCs. This conclusion was further supported by two independent methods: Western blot-based SUnSET assay (Figure 4G) and FACS-based visualization of fluorescently labeled O-Propargyl-puromycin (OP-Puro) incorporation

(B) Heatmap shows differences in abundance of proteins related to carbohydrate, amino acid, and 1C metabolism. $N = 3$, biological replicates (individual cell clones).
(C) Intersection of RNA-seq and proteomics analyses, identifying commonly downregulated genes and corresponding proteins in KO EpiLCs.
(D) Gene ontology enrichment analysis of differentially abundant proteins in Scr and KO EpiLCs, performed using STRING (string-db.org); proteins with log2FC > 0.5 were included in the analysis.
(E) Metabolome analysis of free amino acids in Scr and KO EpiLCs. Colored lines represent different amino acids: green – non-essential, blue – conditionally essential, red - essential; $N = 3$, biological replicates (individual cell clones); error bars indicate SD of the mean; $^{*}p < 0.05$; $^{**}p < 0.01$; and $^{***}p < 0.001$.
(F) Left: Western blot analysis of Scr and KO cells in the ESC and EpiLC states for phosphorylated forms of 4EBP and S6K. Right: quantification analysis of signals from Western blot. $N = 3$, biological replicates (individual cell clones); error bars indicate SD of the mean; $^{**}p < 0.01$ and $^{***}p < 0.001$.
(G) SUnSET assay shows differential protein synthesis rates in Scr and KO cells at the ESC and EpiLC stages.
(H) Normalized protein synthesis rates in Scr and KO cells at the ESC and EpiLC stages revealed by FACS-based visualization of fluorescently labeled O-Propargyl-puromycin (OP-Puro) incorporation. The dashed line represents Scr ESC protein synthesis rate; $N = 3$, biological replicates (individual cell clones); error bars indicate SD of the mean; $^{***}p < 0.001$.

(Figure 4H). Both approaches demonstrated reduced protein synthesis levels in both KO ESCs and KO EpiLCs compared to their Scr counterparts. Notably, these levels closely correlated with the proliferation rates of the cells (Figure 2C), as Scr EpiLCs exhibited an intensification of protein synthesis, whereas KO EpiLCs reached the levels typical of Scr ESCs.

To determine whether the observed phenotype is reversible, we performed a rescue experiment by introducing exogenous *Pcbp1* into the "safe harbor" *Rosa26* locus in *Pcbp1* KO ESCs. Several of the selected clones were found to express exogenous PCBP1 protein (Figure S4E). RT-PCR analysis showed restoration of normal expression levels for both downregulated and upregulated genes in these clones in the EpiLC state (Figure S4F). Consistently, the SUnSET assay demonstrated full recovery of protein synthesis rates in PCBP1-rescued cells (Figure S4G).

Given that *Atf4* mRNA, which encodes a key regulator of amino acid metabolism, was downregulated in KO cells, we tested whether its knockdown could phenocopy the *Pcbp1* KO phenotype. ShRNA-mediated *Atf4* mRNA knockdown led to the reduced expression of its known targets (*Aars1*, *Shmt2*). However, the expression of other genes (e.g., *Got1* and *Slc2a1*), as well as of those upregulated in KO EpiLCs (e.g., *Car2* and *Mup6*), remained unchanged (Figure S4H). Moreover, no comparable decrease in protein synthesis rate (Figure S4I) or proliferation (Figure S4J) was observed. This result suggests that the *Pcbp1* phenotype is partially mediated through ATF4 function; however, the lack of full phenocopy indicates that additional pathways or factors contribute to the full spectrum of effects observed upon loss of PCBP1 function.

Taken together, our results identify PCBP1 as a critical regulator of amino acid metabolism intensification—the process that, in turn, provides protein biosynthesis boost during the naïve-to-primed pluripotency transition. Eventually, this anabolic switch underlies extensive embryo growth after implantation.

DISCUSSION

While the naïve-to-primed pluripotency program switch occurring at and shortly after implantation (Boroviak et al., 2014; Du et al., 2018; Han et al., 2010; Hayashi et al., 2011; Wu et al., 2015; Zhao et al., 2024), including the shift in energy metabolism preference to aerobic glycolysis (Luo et al., 2023a; Zhou et al., 2012), is well studied, little was still known about the rearrangement of anabolic metabolism during this developmental process. In this study, we link, for the first time, the poly(C)-binding protein PCBP1 to amino acid metabolism in pluripotent stem cells during the naïve-to-primed pluripotency transition, providing insight into why PCBP1-deficient embryos die shortly after implantation.

We present clear evidence of PCBP1's role in positively regulating numerous genes associated with amino acid metabolism during the naïve-to-primed pluripotency transition. A deficiency in several amino acids, impaired mTOR activation, along with the downregulation of several tRNA synthetases, appears to lead to a slowdown in protein synthesis, followed by a decline in proliferation rates. Only essential or conditionally essential amino acid levels are dysregulated in PCBP1-deficient cells, which aligns with the downregulation of mRNAs encoding key transporters, including *Slc1a4*, *Slc7a1/3/5/6*, and *Slc3a2*. Elevated glutamine and arginine levels, along with decreased leucine/isoleucine and lysine, may be related to the reduced antiporter activity of *Slc7a5/6* and *Slc3a2* (Gauthier-Coles et al., 2021). The low level of proline appears to be due to the downregulation of *Slc38a1* (Tan et al., 2016), as well as the key enzymes responsible for proline synthesis—*Aldh18a1* and *Pycr* (Phang, 2021). Among DEGs, we also observed *Shmt2*, *Mthfd2*, *Psat1*, *Psph*, and *Phgdh*—genes related to one-carbon (1C) metabolism and serine biosynthesis. These metabolic processes, which are crucial for both proliferation and amino acid metabolism, are typically upregulated in proliferating cells (Altundag and Çelebi-Saltik, 2021; Azad et al., 2023; Shuvalov et al., 2017). 1C metabolism relies on glucose as a substrate, which may explain the downregulation of glycolytic genes despite no detected changes in energy metabolism. Additionally, we demonstrate that ATF4, which transcriptionally regulates all the aforementioned processes—from amino acid uptake and biosynthesis to tRNA charging (Park et al., 2017; Ryan et al., 2021; Selvarajah et al., 2019; Torrence et al., 2021)—is positively regulated by PCBP1. Interestingly, our findings suggest that PCBP1 functions upstream of ATF4 in the regulatory hierarchy controlling amino acid metabolism, as both ATF4 and its targets are downregulated in KO EpiLCs. Moreover, unlike *Pcbp1* KO embryos, ATF4-deficient mice are viable, though they are born with several abnormalities (Masuoka and Townes, 2002). The lethal phenotypes observed in embryos with KOs of amino acid transporters (Poncet et al., 2014; Tsumura et al., 2003), tRNA synthetases (Kalotay et al., 2023), and 1C metabolism genes (Di Pietro et al., 2002) further suggest that ATF4 is not essential for mammalian embryogenesis.

Notably, ATF4 (Lyu and Xu, 2024; Masuoka and Townes, 2002; Zheng et al., 2024), along with PCBP1 and PCBP2 (Ghanem et al., 2015; Ji et al., 2021), has been shown to be important for definitive hematopoiesis. Conditional EpoR-driven Cre-mediated KO of both *Pcbp1* and *Pcbp2* results in the absence of liver erythropoiesis at E12.5 and embryonic lethality at E13.5 (Ji et al., 2021). Interestingly,

the knockout of either *Pcbp1* or *Pcbp2* alone does not lead to lethality in this system, highlighting a unique and essential role for PCBP1 in the naïve-to-primed pluripotency transition. We propose that both of these processes, which are characterized by high proliferation rates (Bowie et al., 2007), require intensified amino acid metabolism, a function dependent on PCBP1.

mTOR signaling plays a central role in transducing growth signals and activating anabolic processes, including protein synthesis (Torrence et al., 2021). In pluripotent cells, mTOR is activated shortly after LIF withdrawal (Cherepkova et al., 2016), while its inhibition supports maintenance of the naive state and can induce diapause (Bulut-Karslioglu et al., 2016; Iyer et al., 2024). Our results show that while 4EBP phosphorylation remains largely unaffected in KO cells, S6K phosphorylation is strongly reduced. A likely explanation is the sensitivity of mTOR signaling to intracellular amino acid levels (Manifava et al., 2016; Sancak et al., 2008), combined with the known differential threshold of 4EBP and S6K to mTOR activity (Choo et al., 2008). We propose that PCBP1, by regulating amino acid import and *de novo* synthesis, supports the intracellular amino acid pool in priming cells. In KO cells, reduced amino acid availability leads to suboptimal mTOR activation, sufficient to phosphorylate 4EBP but not S6K, thereby contributing to decreased protein synthesis rates.

Some findings also suggest a positive role for PCBP1 in tumorigenesis. Extensive research has been conducted on the tumor-related functions of ATF4 (Chen et al., 2022; Du et al., 2021; Wu and Liang, 2024), SLC1A5 (Conger et al., 2024), SLC3A2 (Cano-Crespo et al., 2019), SLC7A5 (Najumudeen et al., 2021), as well as on serine (Geeraerts et al., 2021) and proline biosynthesis (Cui et al., 2023; Westbrook et al., 2022) in cancer. However, reports on PCBP1's role in carcinogenesis are conflicting. While some studies indicate that PCBP1 functions as a tumor suppressor (Chen et al., 2021; Huang et al., 2021; Shi et al., 2018; Thakur et al., 2003; Zhang et al., 2020; Zheng et al., 2022), several articles describe its pro-tumorigenic functions via mRNA stabilization (Peng et al., 2022), TGFβ-signal transduction (Tripathi et al., 2016), or ferroptosis inhibition (Lee et al., 2022; Luo et al., 2023b). However, no studies to date have linked PCBP1 functions in amino acid metabolism to cancer. Interestingly, ATF4 has also been shown to prevent ferroptosis in hepatocellular carcinoma cells (Gao et al., 2021), yet paradoxically, it can also inhibit proliferation and induce apoptosis in tumors (Chen et al., 2024; Zong et al., 2017). Taken together, these results suggest that PCBP1's role in tumorigenesis may be context-dependent, and our results highlight the need for further investigation into its role in amino acid metabolism within this process.

Several open questions remain for future research. Surprisingly, as evidenced by SeaHorse analysis, *Pcbp1* KO cells were able to adapt their energy metabolism to decreased protein biosynthesis, suggesting the existence of a regulatory loop between these two major metabolic processes. We observed a severe downregulation of both RNA and protein levels of GALK1, GOT1, and GPT2 in *Pcbp1* KO cells. While little is known about GALK1's role in pluripotent stem cells, this enzyme participates in galactose catabolism and may contribute to the production of glucose-6P for serine biosynthesis. GPT2 catalyzes the conversion of pyruvate and glutamate into alanine and α-KG, while GOT1 bidirectionally converts asparagine and α-KG into glutamate and oxaloacetate. Interestingly, despite the loss of PCBP1 function, we observed no significant changes in pyruvate and TCA metabolites or different levels of glutamate, alanine, and asparagine. This suggests the presence of compensatory mechanisms that balance amino acid levels, possibly through the inhibition of glutamine conversion to glutamate to prevent excess glutamate accumulation. Finally, the precise mechanism by which PCBP1 regulates amino acid metabolism remains to be elucidated. Our data suggest that PCBP1 plays a role in the transcriptional regulation of the genes mentioned above. Recent studies indicate that PCBP1 stabilizes secondary DNA structures known as i-motifs, thereby preventing the formation of G-quadruplexes on the opposite strand (Karam et al., 2024; Mohanty et al., 2021). However, other functions of PCBP1—such as mRNA stabilization, splicing, and translation—should also be examined in the context of pluripotent stem cells.

RESOURCE AVAILABILITY

Lead contact

Requests for further information and resources should be directed to and will be fulfilled by the lead contact, Evgeny Bakhmet (e.bakhmet@incras.ru).

Materials availability

This study did not generate new unique reagents.

Data and code availability

- The ChIP-seq and RNA-seq datasets have been deposited in the NCBI Gene Expression Omnibus (GEO) database under accession number GSE264037.
- The published ChIP-seq datasets used for comparative analysis are: ATAC-seq (GSE155058), H3K27ac (GSE56098) (Bleckwehl et al., 2021), H3K4me3, H3K9me3, and H3K27me3 (GSE155062) (Buecker et al., 2014).
- The mass spectrometry proteomics data have been deposited in the ProteomeXchange Consortium via the PRIDE (Perez-Riverol et al., 2022) partner repository with the dataset identifier PXD039287.

ACKNOWLEDGMENTS

The study was supported by the Russian Science Foundation grant No. 23-75-10096, https://rscf.ru/https://rscf.ruproject/23-75-10096/. The study was and carried out using the equipment and materials of the Center for Cell Technologies and Vertebrate Cell Culture Collection of the Institute of Cytology (St-Petersburg). Schematic images were created using BioRender.com. The UHPLC-MS/MS analysis was conducted using equipment of the Resource Center for Molecular and Cell Technologies at St-Petersburg State University. We are grateful to Maria V. Igotti for antibodies against 4EBP/p4EBP and S6K/pS6K, and Areti Malapetsas for editing the manuscript.

AUTHOR CONTRIBUTIONS

E.I.B. conceived the study, conducted the majority of the experimental procedures, including KO embryos analysis and cell culture work, wrote the first draft; E.V.P. performed the bioinformatics analysis; O.Y.S. conducted experiments on energy metabolism using a SeaHorse analyzer, measured protein biosynthesis rates, and participated in omics data interpretation; A.A.L. and E.A.R. performed shotgun proteomics and proteomics data analysis, with A.A.L. also contributing to omics data interpretation; D.V.K performed cloning work for Atf4 knockdown and conducted RT-PCR analyses; N.E.V. prepared DNA libraries for ChIP-seq analysis; A.N.K. established *Pcbp1*$^{+/-}$ heterozygous mice; A.S.Z. contributed to cell culture work and molecular cloning; A.A.K. assisted with molecular cloning; N.D.A. performed FACS analysis; A.T.K. carried out metabolome analysis; G.W. conducted experiments with pre-implantation embryos; H.R.S. provided guidance throughout the project and contributed to the writing; and A.N.T. conceived the study, managed the project, contributed to materials, tools, and reagents, edited and approved the final version of the manuscript.

DECLARATION OF INTERESTS

The authors declare no competing interests.

STAR★METHODS

Detailed methods are provided in the online version of this paper and include the following:

- KEY RESOURCES TABLE
- EXPERIMENTAL MODEL AND STUDY PARTICIPANT DETAILS
  - Animals
  - Cell lines
- METHOD DETAILS
  - *Pcbp1* knockdown in preimplantation embryos
  - Generation of *Pcbp1*-heterozygous mice
  - Cell culture work
  - Genetic constructs
  - Cell death analysis
  - RNA extraction and qPCR analysis
  - Immunocytochemical analysis
  - Western blot
  - *In vitro* differentiation
  - Cell cycle analysis
  - Time-lapse microscopy
  - Flow cytometry
  - SeaHorse energy profiling
  - Quantitative comparison of total protein biosynthesis
  - Surface sensing of translation (SUnSET) assay
  - ChIP-seq analysis
  - RNA-seq analysis
  - Shotgun proteomics
  - Metabolomics
- QUANTIFICATION AND STATISTICAL ANALYSIS

SUPPLEMENTAL INFORMATION

Supplemental information can be found online at https://doi.org/10.1016/j.stemcr.2026.102950.

Received: July 1, 2025
Revised: May 6, 2026
Accepted: May 7, 2026
Published: June 4, 2026

REFERENCES

Altundag, Ö., and Çelebi-Saltik, B. (2021). From Embryo to Adult: One Carbon Metabolism in Stem Cells. Curr. Stem Cell Res. Ther. *16*, 175–188. https://doi.org/10.2174/1574888x15666200712191308.

Andrews, S. (2010). FastQC: A Quality Control Tool for High Throughput Sequence Data (Cambridge).

Bae, D.H., Lane, D.J.R., Siafakas, A.R., Sutak, R., Paluncic, J., Huang, M.L.H., Jansson, P.J., Rahmanto, Y.S., and Richardson, D.R. (2020). Acireductone dioxygenase 1 (ADI1) is regulated by cellular iron by a mechanism involving the iron chaperone, PCBP1, with PCBP2 acting as a potential co-chaperone. Biochim. Biophys. Acta. Mol. Basis Dis. *1866*, 165844. https://doi.org/10.1016/j.bbadis.2020.165844.

Bakhmet, E.I., Nazarov, I.B., Artamonova, T.O., Khodorkovsky, M.A., and Tomilin, A.N. (2017). Mass spectrometry for identification of proteins that specifically bind to a distal enhancer of the Oct4 gene. J. Phys, Conf. Ser. *917*, 042027. https://doi.org/10.1088/1742-6596/917/4/042027.

Bakhmet, E.I., Nazarov, I.B., Gazizova, A.R., Vorobyeva, N.E., Kuzmin, A.A., Gordeev, M.N., Sinenko, S.A., Aksenov, N.D., Artamonova, T.O., Khodorkovskii, M.A., et al. (2019). hnRNP-K Targets Open Chromatin in Mouse Embryonic Stem Cells in Concert With Multiple Regulators. Stem Cell. *37*, 1018–1029. https://doi.org/10.1002/stem.3025.

Bakhmet, E.I., Ponomartsev, S.V., Dyban, P.A., Nazarov, I.B., Kuzmin, A.A., Aksenov, N.D., Potapenko, E.V., Gordeev, M.N., and Tomilin, A.N. (2020). Derivation and Characterization of Pcbp1-Deficient Mouse Embryonic Stem Cells. Cell Tissue Biol. *14*, 413–418. https://doi.org/10.1134/s1990519x20060036.

Battulin, N., Kovalzon, V.M., Korablev, A., Serova, I., Kiryukhina, O.O., Pechkova, M.G., Bogotskoy, K.A., Tarasova, O.S., and Panchin, Y. (2021). Pannexin 1 Transgenic Mice: Human Diseases

and Sleep-Wake Function Revision. Int. J. Mol. Sci. *22*, 5269. https://doi.org/10.3390/ijms22105269.

Bleckwehl, T., Crispatzu, G., Schaaf, K., Respuela, P., Bartusel, M., Benson, L., Clark, S.J., Dorighi, K.M., Barral, A., Laugsch, M., et al. (2021). Enhancer-associated H3K4 methylation safeguards in vitro germline competence. Nat. Commun. *12*, 5771. https://doi.org/10.1038/s41467-021-26065-6.

Boroviak, T., Loos, R., Bertone, P., Smith, A., and Nichols, J. (2014). The ability of inner-cell-mass cells to self-renew as embryonic stem cells is acquired following epiblast specification. Nat. Cell Biol. *16*, 516–528. https://doi.org/10.1038/ncb2965.

Bowie, M.B., Kent, D.G., Dykstra, B., McKnight, K.D., McCaffrey, L., Hoodless, P.A., and Eaves, C.J. (2007). Identification of a new intrinsically timed developmental checkpoint that reprograms key hematopoietic stem cell properties. Proc. Natl. Acad. Sci. USA *104*, 5878–5882. https://doi.org/10.1073/pnas.0700460104.

Branden, K.V., and Verboven, S. (2009). Robust data imputation. Comput. Biol. Chem. *33*, 7–13. https://doi.org/10.1016/j.compbiolchem.2008.07.019.

Brons, I.G.M., Smithers, L.E., Trotter, M.W.B., Rugg-Gunn, P., Sun, B., Chuva de Sousa Lopes, S.M., Howlett, S.K., Clarkson, A., Ahrlund-Richter, L., Pedersen, R.A., and Vallier, L. (2007). Derivation of pluripotent epiblast stem cells from mammalian embryos. Nature *448*, 191–195. https://doi.org/10.1038/nature05950.

Buecker, C., Srinivasan, R., Wu, Z., Calo, E., Acampora, D., Faial, T., Simeone, A., Tan, M., Swigut, T., and Wysocka, J. (2014). Reorganization of enhancer patterns in transition from naive to primed pluripotency. Cell Stem Cell *14*, 838–853. https://doi.org/10.1016/j.stem.2014.04.003.

Bulut-Karslioglu, A., Biechele, S., Jin, H., Macrae, T.A., Hejna, M., Gertsenstein, M., Song, J.S., and Ramalho-Santos, M. (2016). Inhibition of mTOR induces a paused pluripotent state. Nature *540*, 119–123. https://doi.org/10.1038/nature20578.

Cano-Crespo, S., Chillarón, J., Junza, A., Fernández-Miranda, G., García, J., Polte, C., R de la Ballina, L., Ignatova, Z., Yanes, Ó., Zorzano, A., et al. (2019). CD98hc (SLC3A2) sustains amino acid and nucleotide availability for cell cycle progression. Sci. Rep. *9*, 14065. https://doi.org/10.1038/s41598-019-50547-9.

Chaudhury, A., Hussey, G.S., Ray, P.S., Jin, G., Fox, P.L., and Howe, P.H. (2010). TGF-beta-mediated phosphorylation of hnRNP E1 induces EMT via transcript-selective translational induction of Dab2 and ILEI. Nat. Cell Biol. *12*, 286–293. https://doi.org/10.1038/ncb2029.

Chen, C., Zhang, Z., Liu, C., Wang, B., Liu, P., Fang, S., Yang, F., You, Y., and Li, X. (2022). ATF4-dependent fructolysis fuels growth of glioblastoma multiforme. Nat. Commun. *13*, 6108. https://doi.org/10.1038/s41467-022-33859-9.

Chen, J., Huang, X., Zhang, S., and Zhu, X. (2024). ATF4 inhibits tumor development and mediates p-GCN2/ASNS upregulation in colon cancer. Sci. Rep. *14*, 13042. https://doi.org/10.1038/s41598-024-63895-y.

Chen, Q., Gu, M., Cai, Z.K., Zhao, H., Sun, S.C., Liu, C., Zhan, M., Chen, Y.B., and Wang, Z. (2021). TGF-beta1 promotes epithelial-to-mesenchymal transition and stemness of prostate cancer cells by inducing PCBP1 degradation and alternative splicing of CD44. Cell. Mol. Life Sci. *78*, 949–962. https://doi.org/10.1007/s00018-020-03544-5.

Chen, S. (2023). Ultrafast one-pass FASTQ data preprocessing, quality control, and deduplication using fastp. iMeta *2*, e107. https://doi.org/10.1002/imt2.107.

Cherepkova, M.Y., Sineva, G.S., and Pospelov, V.A. (2016). Leukemia inhibitory factor (LIF) withdrawal activates mTOR signaling pathway in mouse embryonic stem cells through the MEK/ERK/TSC2 pathway. Cell Death Dis. *7*, e2050. https://doi.org/10.1038/cddis.2015.387.

Choi, J., Huebner, A.J., Clement, K., Walsh, R.M., Savol, A., Lin, K., Gu, H., Di Stefano, B., Brumbaugh, J., Kim, S.Y., et al. (2017). Prolonged Mek1/2 suppression impairs the developmental potential of embryonic stem cells. Nature *548*, 219–223. https://doi.org/10.1038/nature23274.

Choo, A.Y., Yoon, S.O., Kim, S.G., Roux, P.P., and Blenis, J. (2008). Rapamycin differentially inhibits S6Ks and 4E-BP1 to mediate cell-type-specific repression of mRNA translation. Proc. Natl. Acad. Sci. USA *105*, 17414–17419. https://doi.org/10.1073/pnas.0809136105.

Conger, K.O., Chidley, C., Ozgurses, M.E., Zhao, H., Kim, Y., Semina, S.E., Burns, P., Rawat, V., Lietuvninkas, L., Sheldon, R., et al. (2024). ASCT2 is a major contributor to serine uptake in cancer cells. Cell Rep. *43*, 114552. https://doi.org/10.1016/j.celrep.2024.114552.

Cui, B., He, B., Huang, Y., Wang, C., Luo, H., Lu, J., Su, K., Zhang, X., Luo, Y., Zhao, Z., et al. (2023). Pyrroline-5-carboxylate reductase 1 reprograms proline metabolism to drive breast cancer stemness under psychological stress. Cell Death Dis. *14*, 682. https://doi.org/10.1038/s41419-023-06200-5.

Di Pietro, E., Sirois, J., Tremblay, M.L., and MacKenzie, R.E. (2002). Mitochondrial NAD-dependent methylenetetrahydrofolate dehydrogenase-methenyltetrahydrofolate cyclohydrolase is essential for embryonic development. Mol. Cell Biol. *22*, 4158–4166. https://doi.org/10.1128/MCB.22.12.4158-4166.2002.

Dobin, A., Davis, C.A., Schlesinger, F., Drenkow, J., Zaleski, C., Jha, S., Batut, P., Chaisson, M., and Gingeras, T.R. (2013). STAR: ultrafast universal RNA-seq aligner. Bioinformatics *29*, 15–21. https://doi.org/10.1093/bioinformatics/bts635.

Du, J., Liu, H., Mao, X., Qin, Y., and Fan, C. (2021). ATF4 promotes lung cancer cell proliferation and invasion partially through regulating Wnt/beta-catenin signaling. Int. J. Med. Sci. *18*, 1442–1448. https://doi.org/10.7150/ijms.43167.

Du, P., Pirouz, M., Choi, J., Huebner, A.J., Clement, K., Meissner, A., Hochedlinger, K., and Gregory, R.I. (2018). An Intermediate Pluripotent State Controlled by MicroRNAs Is Required for the Naive-to-Primed Stem Cell Transition. Cell Stem Cell *22*, 851–864.e5. https://doi.org/10.1016/j.stem.2018.04.021.

Du, P., and Wu, J. (2024). Hallmarks of totipotent and pluripotent stem cell states. Cell Stem Cell *31*, 312–333. https://doi.org/10.1016/j.stem.2024.01.009.

Freese, N.H., Norris, D.C., and Loraine, A.E. (2016). Integrated genome browser: visual analytics platform for genomics. Bioinformatics *32*, 2089–2095. https://doi.org/10.1093/bioinformatics/btw069.

Gao, R., Kalathur, R.K.R., Coto-Llerena, M., Ercan, C., Buechel, D., Shuang, S., Piscuoglio, S., Dill, M.T., Camargo, F.D., Christofori, G., and Tang, F. (2021). YAP/TAZ and ATF4 drive resistance to Sorafenib in hepatocellular carcinoma by preventing ferroptosis. EMBO Mol. Med. *13*, e14351. https://doi.org/10.15252/emmm.202114351.

Gauthier-Coles, G., Vennitti, J., Zhang, Z., Comb, W.C., Xing, S., Javed, K., Bröer, A., and Bröer, S. (2021). Quantitative modelling of amino acid transport and homeostasis in mammalian cells. Nat. Commun. *12*, 5282. https://doi.org/10.1038/s41467-021-25563-x.

Geeraerts, S.L., Heylen, E., De Keersmaecker, K., and Kampen, K.R. (2021). The ins and outs of serine and glycine metabolism in cancer. Nat. Metab. *3*, 131–141. https://doi.org/10.1038/s42255-020-00329-9.

Ghanem, L.R., Kromer, A., Silverman, I.M., Chatterji, P., Traxler, E., Penzo-Mendez, A., Weiss, M.J., Stanger, B.Z., and Liebhaber, S.A. (2016). The Poly(C) Binding Protein Pcbp2 and Its Retrotransposed Derivative Pcbp1 Are Independently Essential to Mouse Development. Mol. Cell Biol. *36*, 304–319. https://doi.org/10.1128/MCB.00936-15.

Goodman, C.A., and Hornberger, T.A. (2013). Measuring protein synthesis with SUnSET: a valid alternative to traditional techniques? Exerc. Sport Sci. Rev. *41*, 107–115. https://doi.org/10.1097/JES.0b013e3182798a95.

Guo, G., Yang, J., Nichols, J., Hall, J.S., Eyres, I., Mansfield, W., and Smith, A. (2009). Klf4 reverts developmentally programmed restriction of ground state pluripotency. Development *136*, 1063–1069. https://doi.org/10.1242/dev.030957.

Han, D.W., Tapia, N., Joo, J.Y., Greber, B., Araúzo-Bravo, M.J., Bernemann, C., Ko, K., Wu, G., Stehling, M., Do, J.T., and Schöler, H.R. (2010). Epiblast stem cell subpopulations represent mouse embryos of distinct pregastrulation stages. Cell *143*, 617–627. https://doi.org/10.1016/j.cell.2010.10.015.

Hayashi, K., Ohta, H., Kurimoto, K., Aramaki, S., and Saitou, M. (2011). Reconstitution of the mouse germ cell specification pathway in culture by pluripotent stem cells. Cell *146*, 519–532. https://doi.org/10.1016/j.cell.2011.06.052.

Huang, S., Luo, K., Jiang, L., Zhang, X.D., Lv, Y.H., and Li, R.F. (2021). PCBP1 regulates the transcription and alternative splicing of metastasisrelated genes and pathways in hepatocellular carcinoma. Sci. Rep. *11*, 23356. https://doi.org/10.1038/s41598-021-02642-z.

Huang, X., Balmer, S., Yang, F., Fidalgo, M., Li, D., Guallar, D., Hadjantonakis, A.K., and Wang, J. (2017). Zfp281 is essential for mouse epiblast maturation through transcriptional and epigenetic control of Nodal signaling. eLife *6*, e33333. https://doi.org/10.7554/eLife.33333.

Hussey, G.S., Chaudhury, A., Dawson, A.E., Lindner, D.J., Knudsen, C.R., Wilce, M.C.J., Merrick, W.C., and Howe, P.H. (2011). Identification of an mRNP complex regulating tumorigenesis at the translational elongation step. Mol. Cell *41*, 419–431. https://doi.org/10.1016/j.molcel.2011.02.003.

Ishii, T., Igawa, T., Hayakawa, H., Fujita, T., Sekiguchi, M., and Nakabeppu, Y. (2020). PCBP1 and PCBP2 both bind heavily oxidized RNA but cause opposing outcomes, suppressing or increasing apoptosis under oxidative conditions. J. Biol. Chem. *295*, 12247–12261. https://doi.org/10.1074/jbc.RA119.011870.

Iyer, D.P., Khoei, H.H., van der Weijden, V.A., Kagawa, H., Pradhan, S.J., Novatchkova, M., McCarthy, A., Rayon, T., Simon, C.S., Dunkel, I., et al. (2024). mTOR activity paces human blastocyst stage developmental progression. Cell *187*, 6566–6583.e22. https://doi.org/10.1016/j.cell.2024.08.048.

Ji, X., Jha, A., Humenik, J., Ghanem, L.R., Kromer, A., Duncan-Lewis, C., Traxler, E., Weiss, M.J., Barash, Y., and Liebhaber, S.A. (2021). RNA-binding proteins PCBP1 and PCBP2 are critical determinants of murine erythropoiesis. Mol. Cell Biol. *41*, MCB0066820. https://doi.org/10.1128/MCB.00668-20.

Kalotay, E., Klugmann, M., Housley, G.D., and Fröhlich, D. (2023). Recessive aminoacyl-tRNA synthetase disorders: lessons learned from in vivo disease models. Front. Neurosci. *17*, 1182874. https://doi.org/10.3389/fnins.2023.1182874.

Karam, J.A.Q., Fréreux, C., Mohanty, B.K., Dalton, A.C., Dincman, T.A., Palanisamy, V., Howley, B.V., and Howe, P.H. (2024). The RNA-binding protein PCBP1 modulates transcription by recruiting the G-quadruplex-specific helicase DHX9. J. Biol. Chem. *300*, 107830. https://doi.org/10.1016/j.jbc.2024.107830.

Kinoshita, M., Barber, M., Mansfield, W., Cui, Y., Spindlow, D., Stirparo, G.G., Dietmann, S., Nichols, J., and Smith, A. (2021). Capture of Mouse and Human Stem Cells with Features of Formative Pluripotency. Cell Stem Cell *28*, 453–471.e8. https://doi.org/10.1016/j.stem.2020.11.005.

Kong, A.T., Leprevost, F.V., Avtonomov, D.M., Mellacheruvu, D., and Nesvizhskii, A.I. (2017). MSFragger: ultrafast and comprehensive peptide identification in mass spectrometry-based proteomics. Nat. Methods *14*, 513–520. https://doi.org/10.1038/nmeth.4256.

Krapacher, F.A., Fernández-Suárez, D., Andersson, A., Carrier-Ruiz, A., and Ibáñez, C.F. (2022). Convergent dopamine and ALK4 signaling to PCBP1 controls FosB alternative splicing and cocaine behavioral sensitization. EMBO J. *41*, e110721. https://doi.org/10.15252/embj.2022110721.

Kuzmin, A.A., Ermakova, V.V., Sinenko, S.A., Ponomartsev, S.V., Starkova, T.Y., Skvortsova, E.V., Cherepanova, O., and Tomilin, A.N. (2019). Genetic tool for fate mapping of Oct4 (Pou5f1)-expressing cells and their progeny past the pluripotency stage. Stem Cell Res. Ther. *10*, 391. https://doi.org/10.1186/s13287-019-1520-6.

Lando, D., Ma, X., Cao, Y., Jartseva, A., Stevens, T.J., Boucher, W., Reynolds, N., Montibus, B., Hall, D., Lackner, A., et al. (2024). Enhancer-promoter interactions are reconfigured through the formation of long-range multiway hubs as mouse ES cells exit pluripotency. Mol. Cell *84*, 1406–1421.e8. https://doi.org/10.1016/j.molcel.2024.02.015.

Langmead, B., and Salzberg, S.L. (2012). Fast gapped-read alignment with Bowtie 2. Nat. Methods *9*, 357–359. https://doi.org/10.1038/nmeth.1923.

Lee, J., You, J.H., and Roh, J.-L. (2022). Poly(rC)-binding protein 1 represses ferritinophagy-mediated ferroptosis in head and neck cancer. Redox Biol. *51*, 102276. https://doi.org/10.1016/j.redox.2022.102276.

Liu, T. (2014). Use model-based Analysis of ChIP-Seq (MACS) to analyze short reads generated by sequencing protein-DNA interactions in embryonic stem cells. Methods Mol. Biol. *1150*, 81. https://doi.org/10.1007/978-1-4939-0512-6_4.

Love, M.I., Huber, W., and Anders, S. (2014). Moderated estimation of fold change and dispersion for RNA-seq data with DESeq2. Genome Biol. *15*, 550. https://doi.org/10.1186/s13059-014-0550-8.

Luo, Q., Pui, H.P., Chen, J., Yu, L., Jannig, P.R., Pei, Y., Zhao, L., Chen, X., Petropoulos, S., Ruas, J.L., et al. (2023a). Epiblast-like stem cells established by Wnt/beta-catenin signaling manifest distinct features of formative pluripotency and germline competence. Cell Rep. *42*, 112021. https://doi.org/10.1016/j.celrep.2023.112021.

Luo, Y., Zhang, Y., Pang, S., Min, J., Wang, T., Wu, D., Lin, C., Xiao, Z., Xiang, Q., Li, Q., and Ma, L. (2023b). PCBP1 protects bladder cancer cells from mitochondria injury and ferroptosis by inducing LACTB mRNA degradation. Mol. Carcinog. *62*, 907–919. https://doi.org/10.1002/mc.23533.

Lyu, J., and Xu, J. (2024). Context matters: role of ATF4 in hematopoiesis. Blood *144*, 684–686. https://doi.org/10.1182/blood.2024025066.

Manifava, M., Smith, M., Rotondo, S., Walker, S., Niewczas, I., Zoncu, R., Clark, J., and Ktistakis, N.T. (2016). Dynamics of mTORC1 activation in response to amino acids. eLife *5*, e19960. https://doi.org/10.7554/eLife.19960.

Masuoka, H.C., and Townes, T.M. (2002). Targeted disruption of the activating transcription factor 4 gene results in severe fetal anemia in mice. Blood *99*, 736–745. https://doi.org/10.1182/blood.v99.3.736.

Mathieu, J., and Ruohola-Baker, H. (2017). Metabolic remodeling during the loss and acquisition of pluripotency. Development *144*, 541–551. https://doi.org/10.1242/dev.128389.

Mirzadeh Azad, F., Struys, E.A., Wingert, V., Hannibal, L., Mills, K., Jansen, J.H., Longley, D.B., Stunnenberg, H.G., and Atlasi, Y. (2023). Spic regulates one-carbon metabolism and histone methylation in ground-state pluripotency. Sci. Adv. *9*, eadg7997. https://doi.org/10.1126/sciadv.adg7997.

Mohanty, B.K., Karam, J.A., Howley, B.V., Dalton, A.C., Grelet, S., Dincman, T., Streitfeld, W.S., Yoon, J.H., Balakrishnan, L., Chazin, W.J., et al. (2021). Heterogeneous nuclear ribonucleoprotein E1 binds polycytosine DNA and monitors genome integrity. Life Sci. Alliance *4*, e202000995. https://doi.org/10.26508/lsa.202000995.

Najumudeen, A.K., Ceteci, F., Fey, S.K., Hamm, G., Steven, R.T., Hall, H., Nikula, C.J., Dexter, A., Murta, T., Race, A.M., et al. (2021). The amino acid transporter SLC7A5 is required for efficient growth of KRAS-mutant colorectal cancer. Nat. Genet. *53*, 16–26. https://doi.org/10.1038/s41588-020-00753-3.

Park, Y., Reyna-Neyra, A., Philippe, L., and Thoreen, C.C. (2017). mTORC1 Balances Cellular Amino Acid Supply with Demand for Protein Synthesis through Post-transcriptional Control of ATF4. Cell Rep. *19*, 1083–1090. https://doi.org/10.1016/j.celrep.2017.04.042.

Patel, S.J., Frey, A.G., Palenchar, D.J., Achar, S., Bullough, K.Z., Vashisht, A., Wohlschlegel, J.A., and Philpott, C.C. (2019). A PCBP1-BolA2 chaperone complex delivers iron for cytosolic [2Fe-2S] cluster assembly. Nat. Chem. Biol. *15*, 872–881. https://doi.org/10.1038/s41589-019-0330-6.

Peng, K., Chen, X., Lin, A., Tong, Z., and Lin, W. (2022). PolyC-RNA-binding protein 1 (PCBP1) enhances tropomyosin 3 (TPM3) mRNA stability to promote the progression of esophageal squamous cell carcinoma. Bioengineered *13*, 8581–8592. https://doi.org/10.1080/21655979.2022.2053801.

Pera, M.F., and Rossant, J. (2021). The exploration of pluripotency space: Charting cell state transitions in peri-implantation development. Cell Stem Cell *28*, 1896–1906. https://doi.org/10.1016/j.stem.2021.10.001.

Perez-Riverol, Y., Bai, J., Bandla, C., Garcia-Seisdedos, D., Hewapathirana, S., Kamatchinathan, S., Kundu, D.J., Prakash, A., Frericks-Zipper, A., Eisenacher, M., et al. (2022). The PRIDE database resources in 2022: a hub for mass spectrometry-based proteomics evidences. Nucleic acids research *50*, D543–D552. https://doi.org/10.1093/nar/gkab1038.

Phang, J.M. (2021). Perspectives, past, present and future: the proline cycle/proline-collagen regulatory axis. Amino Acids *53*, 1967–1975. https://doi.org/10.1007/s00726-021-03103-7.

Poncet, N., Mitchell, F.E., Ibrahim, A.F.M., McGuire, V.A., English, G., Arthur, J.S.C., Shi, Y.B., and Taylor, P.M. (2014). The catalytic subunit of the system L1 amino acid transporter (slc7a5) facilitates nutrient signalling in mouse skeletal muscle. PLoS One *9*, e89547. https://doi.org/10.1371/journal.pone.0089547.

Ritchie, M.E., Phipson, B., Wu, D., Hu, Y., Law, C.W., Shi, W., and Smyth, G.K. (2015). limma powers differential expression analyses for RNA-sequencing and microarray studies. Nucleic Acids Res. *43*, e47. https://doi.org/10.1093/nar/gkv007.

Ryan, D.G., Yang, M., Prag, H.A., Blanco, G.R., Nikitopoulou, E., Segarra-Mondejar, M., Powell, C.A., Young, T., Burger, N., Miljkovic, J.L., et al. (2021). Disruption of the TCA cycle reveals an ATF4-dependent integration of redox and amino acid metabolism. eLife *10*, e72593. https://doi.org/10.7554/eLife.72593.

Sancak, Y., Peterson, T.R., Shaul, Y.D., Lindquist, R.A., Thoreen, C.C., Bar-Peled, L., and Sabatini, D.M. (2008). The Rag GTPases bind raptor and mediate amino acid signaling to mTORC1. Science *320*, 1496–1501. https://doi.org/10.1126/science.1157535.

Santini, L., Kowald, S., Cerron-Alvan, L.M., Huth, M., Fabing, A.P., Sestini, G., Rivron, N., and Leeb, M. (2024). FoxO transcription factors actuate the formative pluripotency specific gene expression programme. Nat. Commun. *15*, 7879. https://doi.org/10.1038/s41467-024-51794-9.

Selvarajah, B., Azuelos, I., Platé, M., Guillotin, D., Forty, E.J., Contento, G., Woodcock, H.V., Redding, M., Taylor, A., Brunori, G., et al. (2019). mTORC1 amplifies the ATF4-dependent de novo serine-glycine pathway to supply glycine during TGF-β(1)-induced collagen biosynthesis. Sci. Signal. *12*, eaav3048. https://doi.org/10.1126/scisignal.aav3048.

Shi, H., Li, H., Yuan, R., Guan, W., Zhang, X., Zhang, S., Zhang, W., Tong, F., Li, L., Song, Z., et al. (2018). PCBP1 depletion promotes tumorigenesis through attenuation of p27(Kip1) mRNA stability and translation. J. Exp. Clin. Cancer Res. *37*, 187. https://doi.org/10.1186/s13046-018-0840-1.

Shuvalov, O., Petukhov, A., Daks, A., Fedorova, O., Vasileva, E., and Barlev, N.A. (2017). One-carbon metabolism and nucleotide biosynthesis as attractive targets for anticancer therapy. Oncotarget *8*, 23955–23977. https://doi.org/10.18632/oncotarget.15053.

Sugimoto, M., Kondo, M., Koga, Y., Shiura, H., Ikeda, R., Hirose, M., Ogura, A., Murakami, A., Yoshiki, A., Chuva de Sousa Lopes, S.M., and Abe, K. (2015). A simple and robust method for establishing homogeneous mouse epiblast stem cell lines by wnt inhibition. Stem Cell Rep. *4*, 744–757. https://doi.org/10.1016/j.stemcr.2015.02.014.

Sumi, T., Oki, S., Kitajima, K., and Meno, C. (2013). Epiblast ground state is controlled by canonical Wnt/beta-catenin signaling in the postimplantation mouse embryo and epiblast stem cells. PLoS One *8*, e63378. https://doi.org/10.1371/journal.pone.0063378.

Szabó, P.E., Hübner, K., Schöler, H., and Mann, J.R. (2002). Allele-specific expression of imprinted genes in mouse migratory primordial germ cells. Mech. Dev. *115*, 157–160. https://doi.org/10.1016/s0925-4773(02)00087-4.

Szklarczyk, D., Gable, A.L., Nastou, K.C., Lyon, D., Kirsch, R., Pyysalo, S., Doncheva, N.T., Legeay, M., Fang, T., Bork, P., et al. (2021). The STRING database in 2021: customizable protein-protein networks, and functional characterization of user-uploaded gene/measurement sets. Nucleic Acids Res. *49*, D605–D612. https://doi.org/10.1093/nar/gkaa1074.

Takeo, T., and Nakagata, N. (2018). In Vitro Fertilization in Mice. Cold Spring Harb. Protoc. *2018*. https://doi.org/10.1101/pdb.prot094524.

Tan, B.S.N., Rathjen, P.D., Harvey, A.J., Gardner, D.K., and Rathjen, J. (2016). Regulation of amino acid transporters in pluripotent cell populations in the embryo and in culture; novel roles for sodium-coupled neutral amino acid transporters. Mech. Dev. *141*, 32. https://doi.org/10.1016/j.mod.2016.06.003.

Tesar, P.J., Chenoweth, J.G., Brook, F.A., Davies, T.J., Evans, E.P., Mack, D.L., Gardner, R.L., and McKay, R.D.G. (2007). New cell lines from mouse epiblast share defining features with human embryonic stem cells. Nature *448*, 196–199. https://doi.org/10.1038/nature05972.

Thakur, S., Nakamura, T., Calin, G., Russo, A., Tamburrino, J.F., Shimizu, M., Baldassarre, G., Battista, S., Fusco, A., Wassell, R.P., et al. (2003). Regulation of BRCA1 Transcription by Specific Single-Stranded DNA Binding Factors. Mol. Cell Biol. *23*, 3774–3787. https://doi.org/10.1128/mcb.23.11.3774-3787.2003.

Torrence, M.E., MacArthur, M.R., Hosios, A.M., Valvezan, A.J., Asara, J.M., Mitchell, J.R., and Manning, B.D. (2021). The mTORC1-mediated activation of ATF4 promotes protein and glutathione synthesis downstream of growth signals. eLife *10*, e63326. https://doi.org/10.7554/eLife.63326.

Tosolini, M., and Jouneau, A. (2016). From Naive to Primed Pluripotency: In Vitro Conversion of Mouse Embryonic Stem Cells in Epiblast Stem Cells. Methods Mol. Biol. *1341*, 209–216. https://doi.org/10.1007/7651_2015_208.

Tripathi, V., Sixt, K.M., Gao, S., Xu, X., Huang, J., Weigert, R., Zhou, M., and Zhang, Y.E. (2016). Direct Regulation of Alternative Splicing by SMAD3 through PCBP1 Is Essential to the Tumor-Promoting Role of TGF-beta. Mol. Cell *64*, 549–564. https://doi.org/10.1016/j.molcel.2016.09.013.

Tsumura, H., Suzuki, N., Saito, H., Kawano, M., Otake, S., Kozuka, Y., Komada, H., Tsurudome, M., and Ito, Y. (2003). The targeted disruption of the CD98 gene results in embryonic lethality. Biochem. Biophys. Res. Commun. *308*, 847–851. https://doi.org/10.1016/s0006-291x(03)01473-6.

Van Nostrand, E.L., Freese, P., Pratt, G.A., Wang, X., Wei, X., Xiao, R., Blue, S.M., Chen, J.Y., Cody, N.A.L., Dominguez, D., et al. (2020). A large-scale binding and functional map of human RNA-binding proteins. Nature *583*, 711–719. https://doi.org/10.1038/s41586-020-2077-3.

Wang, J., Zhang, Y., Gao, J., Feng, G., Liu, C., Li, X., Li, P., Liu, Z., Lu, F., Wang, L., et al. (2024). Alternative splicing of CARM1 regulated by LincGET-guided paraspeckles biases the first cell fate in mammalian early embryos. Nat. Struct. Mol. Biol. *31*, 1341–1354. https://doi.org/10.1038/s41594-024-01292-9.

Wang, L., Wang, S., and Li, W. (2012). RSeQC: quality control of RNA-seq experiments. Bioinformatics *28*, 2184–2185. https://doi.org/10.1093/bioinformatics/bts356.

Wang, Q., Li, M., Wu, T., Zhan, L., Li, L., Chen, M., Xie, W., Xie, Z., Hu, E., Xu, S., and Yu, G. (2022). Exploring Epigenomic Datasets by ChIPseeker. Curr. Protoc. *2*, e585. https://doi.org/10.1002/cpz1.585.

Wang, S., Li, W., Hu, L., Cheng, J., Yang, H., and Liu, Y. (2020). NAguideR: performing and prioritizing missing value imputations for consistent bottom-up proteomic analyses. Nucleic Acids Res. *48*, e83. https://doi.org/10.1093/nar/gkaa498.

Wang, Y., Protchenko, O., Huber, K.D., Shakoury-Elizeh, M., Ghosh, M.C., and Philpott, C.C. (2023). The iron chaperone poly(rC)-binding protein 1 regulates iron efflux through intestinal ferroportin in mice. Blood *142*, 1658–1671. https://doi.org/10.1182/blood.2023020504.

Weinberger, L., Ayyash, M., Novershtern, N., and Hanna, J.H. (2016). Dynamic stem cell states: naive to primed pluripotency in rodents and humans. Nat. Rev. Mol. Cell Biol. *17*, 155–169. https://doi.org/10.1038/nrm.2015.28.

Westbrook, R.L., Bridges, E., Roberts, J., Escribano-Gonzalez, C., Eales, K.L., Vettore, L.A., Walker, P.D., Vera-Siguenza, E., Rana, H., Cuozzo, F., et al. (2022). Proline synthesis through PYCR1 is required to support cancer cell proliferation and survival in oxygen-limiting conditions. Cell Rep. *38*, 110320. https://doi.org/10.1016/j.celrep.2022.110320.

Wu, D., and Liang, J. (2024). Activating transcription factor 4: a regulator of stress response in human cancers. Front. Cell Dev. Biol. *12*, 1370012. https://doi.org/10.3389/fcell.2024.1370012.

Wu, J., Okamura, D., Li, M., Suzuki, K., Luo, C., Ma, L., He, Y., Li, Z., Benner, C., Tamura, I., et al. (2015). An alternative pluripotent state confers interspecies chimaeric competency. Nature *521*, 316–321. https://doi.org/10.1038/nature14413.

Wu, T., Hu, E., Xu, S., Chen, M., Guo, P., Dai, Z., Feng, T., Zhou, L., Tang, W., Zhan, L., et al. (2021). clusterProfiler 4.0: A universal enrichment tool for interpreting omics data. Innovation *2*, 100141. https://doi.org/10.1016/j.xinn.2021.100141.

Yagi, M., Kishigami, S., Tanaka, A., Semi, K., Mizutani, E., Wakayama, S., Wakayama, T., Yamamoto, T., and Yamada, Y. (2017).

Derivation of ground-state female ES cells maintaining gamete-derived DNA methylation. Nature *548*, 224–227. https://doi.org/10.1038/nature23286.

Zhang, J.P., Li, X.L., Li, G.H., Chen, W., Arakaki, C., Botimer, G.D., Baylink, D., Zhang, L., Wen, W., Fu, Y.W., et al. (2017). Efficient precise knockin with a double cut HDR donor after CRISPR/Cas9-mediated double-stranded DNA cleavage. Genome Biol. *18*, 35. https://doi.org/10.1186/s13059-017-1164-8.

Zhang, X., Di, C., Chen, Y., Wang, J., Su, R., Huang, G., Xu, C., Chen, X., Long, F., Yang, H., and Zhang, H. (2020). Multilevel regulation and molecular mechanism of poly (rC)-binding protein 1 in cancer. FASEB J. *34*, 15647–15658. https://doi.org/10.1096/fj.202000911R.

Zhao, B., Yu, X., Shi, J., Ma, S., Li, S., Shi, H., Xia, S., Ye, Y., Zhang, Y., Du, Y., and Wang, Q. (2024). A stepwise mode of TGFbeta-SMAD signaling and DNA methylation regulates naive-to-primed pluripotency and differentiation. Nat. Commun. *15*, 10123. https://doi.org/10.1038/s41467-024-54433-5.

Zheng, Y., Zhou, Z., Wei, R., Xiao, C., Zhang, H., Fan, T., Zheng, B., Li, C., and He, J. (2022). The RNA-binding protein PCBP1 represses lung adenocarcinoma progression by stabilizing DKK1 mRNA and subsequently downregulating beta-catenin. J. Transl. Med. *20*, 343. https://doi.org/10.1186/s12967-022-03552-y.

Zheng, Z., Yang, S., Gou, F., Tang, C., Zhang, Z., Gu, Q., Sun, G., Jiang, P., Wang, N., Zhao, X., et al. (2024). The ATF4-RPS19BP1 axis modulates ribosome biogenesis to promote erythropoiesis. Blood *144*, 742–756. https://doi.org/10.1182/blood.2023021901.

Zhou, W., Choi, M., Margineantu, D., Margaretha, L., Hesson, J., Cavanaugh, C., Blau, C.A., Horwitz, M.S., Hockenbery, D., Ware, C., and Ruohola-Baker, H. (2012). HIF1alpha induced switch from bivalent to exclusively glycolytic metabolism during ESC-to-EpiSC/hESC transition. EMBO J. *31*, 2103–2116. https://doi.org/10.1038/emboj.2012.71.

Zong, Y., Feng, S., Cheng, J., Yu, C., and Lu, G. (2017). Up-Regulated ATF4 Expression Increases Cell Sensitivity to Apoptosis in Response to Radiation. Cell. Physiol. Biochem. *41*, 784–794. https://doi.org/10.1159/000458742.

STAR★METHODS

KEY RESOURCES TABLE

REAGENT or RESOURCE	SOURCE	IDENTIFIER
**Antibodies**		
Pcbp1	Abcam	Cat# ab74793; RRID: AB_1281060
Oct4	Santa Cruz	Cat# sc-5279 (C-10); RRID: AB_628051
Nanog	Bethyl	Cat# A300-397A; RRID: AB_386108
Oct6	Abcam	Cat# ab272925; RRID: AB_2927579
Foxa2 (HNF-3β)	Santa Cruz	Cat# sc-374375; RRID: AB_10989476
Sox17	R&D Systems	Cat# AF1924; RRID: AB_355060
Klf4	Abcam	Cat# ab129473; RRID: AB_2941800
Tuj1	Covance	Cat# MMS-435P; RRID: AB_2315514
α-SMA	Sigma	Cat# A2547; RRID: AB_476701
4E-BP	Cell Signaling	Cat# 9452; RRID: AB_331692
Phospho-4E-BP1 (Thr37/46)	Cell Signaling	Cat# 9459; RRID: AB_330985
S6K	Cell Signaling	Cat# 2317; RRID: AB_2238583
Phospho-S6 (Ser235/236)	Cell Signaling	Cat# 2211; RRID: AB_331679
Gapdh	Cell Signaling	Cat# 2118; RRID: AB_561053
β-Actin	Cell Signaling	Cat# 8457; RRID: AB_10950489
Annexin V	Invitrogen	Cat# A23204; RRID: AB_2341149
Puromycin	Merck	Cat# MABE 343; RRID: AB_2566826
**Chemicals, peptides, and recombinant proteins**		
Leukemia inhibitory factor (LIF)	Made in house	N/A
PD0325901	Axon Medchem	Cat#1408
CHIR99021	Axon Medchem	Cat#1386
XAV939	Sigma Aldrich	Cat#X3004
BMS 493	Sigma Aldrich	Cat#B6688
Basic Fgf (Fgf2)	Peprotech	Cat#100-18C
Activin A	Peprotech	Cat#120-14E
Knockout DMEM	Gibco	Cat#10829018
DMEM/F12 + GlutaMAX Medium	Gibco	Cat#10565018
Neurobasal Medium	Gibco	Cat#21103049
MEM non-essential amino acids (100X)	Gibco	Cat#11140050
GlutaMAX (100X)	Gibco	Cat#35050061
Pen Strep (100X)	Gibco	Cat#15140122

(*Continued on next page*)

Continued

REAGENT or RESOURCE	SOURCE	IDENTIFIER
N-2 Supplement (100X)	Gibco	Cat#17502048
B-27 Supplement (50X)	Gibco	Cat#17504044
FuGENE HD Transfection Reagent	Promega	Cat#E2311
Human Fibronectin Protein	R&D systems	Cat#1918-FN
Poly-L-ornithine hydrobromide	Sigma Aldrich	Cat#P3655
Fetal Bovine Serum	Cytiva	Cat#SV30160.03
MabSelect SuRe	Sigma Aldrich	Cat#GE17547401
**Critical commercial assays**		
Seahorse XF Real-Time ATP Rate Assay Kit	Agilent	Cat#103591
Protein Synthesis Assay Kit	abcam	Cat#ab239725
**Deposited data**		
Raw and analyzed RNA-seq and ChIP-seq data	This study	GSE264037
Raw and analyzed mass spectrometry proteomics data	This study	PXD039287
External re-analysed ATAC-seq	(Bleckwehl et al., 2021)	GSE155058
External re-analysed ChIP-seq for H3K27ac	(Buecker et al., 2014)	GSE56098
External re-analysed ChIP-seq for H3K4me3, H3K9me3, and H3K27me3	(Bleckwehl et al., 2021)	GSE155062
**Experimental models: Cell lines**		
Mus musculus: Embryonic stem cells E14 Tg2a (male)	ATCC	CRL-1821
E14 Tg2a control cell lines “Scr1”, “Scr2”, “Scr3”	(Bakhmet et al., 2020)	N/A
E14 Tg2a Pcbp1-null cell lines “KO1”, “KO2”, “KO3”	(Bakhmet et al., 2020)	N/A
E14 Tg2a KO1 Pcbp1-rescued cell lines “R2”, “R9”, “R12”	This study	N/A
E14 Tg2a “shScr” and “sh-Atf4” with downregulated Atf4 gene expression	This study	N/A
**Experimental models: Organisms/strains**		
Mouse: B6;CBA-Tg(Pou5f1-EGFP)2Mnn/J (OG2)	The Jackson Laboratory	JAX: 004654
Mouse: C57BL/6J	The Jackson Laboratory	JAX:000664
Mouse: CD-1	Chirles River	Strain Code: 022
**Oligonucleotides**		
See Tables S2, S3, S4 and S5 for oligonucleotides used in this study	N/A	N/A
**Software and algorithms**		
Prism v10.1.1	GraphPad	RRID: SCR_002798
ModFit LT 3.0 software	Verity Software House	RRID: SCR_016106
R v4.2.1	R Core Team	RRID: SCR_001905
fastp v0.23.2	(Chen, 2023)	RRID: SCR_016962
FastQC v0.11.9	(Andrews, 2010)	RRID: SCR_014583
Bowtie2 v2.4.5	(Langmead and Salzberg, 2012)	RRID: SCR_016368

(*Continued on next page*)

Continued

REAGENT or RESOURCE	SOURCE	IDENTIFIER
Picard v2.27.4	Broad Institute	RRID: SCR_006525
MACS2 v2.2.7.1	(Liu, 2014)	RRID: SCR_013291
ChIPseeker v1.32.0	(Wang et al., 2022)	RRID: SCR_021322
STAR v2.7.10a	(Dobin et al., 2013)	RRID: SCR_004463
RSeQC v4.0.0	(Wang et al., 2012)	RRID: SCR_005275
DESeq2 v1.36.0	(Love et al., 2014)	RRID: SCR_015687
clusterProfiler v4.4.0	(Wu et al., 2021)	RRID: SCR_016884
Gene Ontology	Gene Ontology Consortium	RRID: SCR_002811
FragPipe (v. 17.1)	(Kong et al., 2017)	RRID: SCR_022864
Integrated Genome Browser v10.2.0	(Freese et al., 2016)	RRID: SCR_011792

EXPERIMENTAL MODEL AND STUDY PARTICIPANT DETAILS

Animals

For *Pcbp1* knockdown in preimplantation embryos 4-10 week-old OG2 mice (male/female) were bred and maintained in the animal facilities of Max Planck Institute for Molecular Biomedicine under pathogen-free conditions on a 12-h light/12-h dark cycle at 23°C–25°C. Animal studies were performed in accordance with the German Animal Welfare guidelines and approved by the Landesamt für Natur, Umwelt und Verbraucherschutz Nordrhein-Westfalen (IACUC protocol 84–02.04.2014.A239).

For generation of *Pcbp1*-heterozygous mice 4–5-week-old C57BL/6J female mice, 10-12-week-old C57BL/6J males, 6-8 week-old outbred CD-1 females, and 8-10 week-old outbred CD-1 males were used. All animals were maintained in the SPF Animal Facility of the Institute of Cytology and Genetics SB RAS (Novosibirsk, Russia). Female mice were housed in groups of four to five per cage (Optimice, Animal Care Systems, Centennial, CO, USA). C57BL/6J and CD-1 male mice were housed in groups of two to three per cage; vasectomized CD-1 males were housed individually. Animals were kept under controlled environmental conditions at 24 ± 2°C, 45%–50% relative humidity, and a 14:10 h light–dark cycle. Food and water were provided *ad libitum*. All animal procedures and technical manipulations were performed in compliance with the European Communities Council Directive of 24 November 1986 (86/609/EEC) and were approved by the Bioethical Committee at the Institute of Cytology and Genetics (Permission N45 from 16 November 2018).

Cell lines

Murine embryonic stem cell (ESC) mutants used in this study were derived from Tg2a E14 ESCs (male, derived from 129/Ola blastocyst). Control cell lines (Scr1, Scr2, Scr3), and *Pcbp1*-knockout lines with *Pcbp1* ORF shift (KO1, KO3, and KO22) were previously reported (Bakhmet et al., 2020). *Mthfd2*-T2A-EGFP Scr/KO ESC lines, as well as sh-*Atf4* ESCs were derived from Tg2a E14 ESCs. All cell lines were routinely tested to ensure that they were not contaminated with mycoplasma.

METHOD DETAILS

Pcbp1 knockdown in preimplantation embryos

Fertilized oocytes were collected in M2 medium 18 h post-hCG from the oviducts of primed OG2 female mice after mating with OG2 male mice. The oocytes were cultured in KSOM medium at 37°C in a 5% CO_2 atmosphere until microinjection. Three siRNA duplex oligonucleotides targeting *Pcbp1* mRNA were tested (Table S1). The lyophilized siRNA duplexes (Dharmacon, Lafayette, CO) were resuspended in siRNA buffer (Dharmacon, cat. no. B-002000-UB-100) according to the manufacturer's instructions and stored in single-use aliquots at −20°C. Microinjection of siRNAs was performed using a FemtoJet microinjector (Eppendorf) and a micromanipulator (Narishige) in M2 medium drops covered with mineral oil. Microinjection pipettes were pulled using a Sutter P-97 pipette puller. Five microliters of siRNA (20 μM) were loaded

into the pipette and approximately 2 pL of siRNA solution were injected into the cytoplasm of each oocyte. Following microinjection, oocytes were washed and cultured in KSOM medium at 37°C in a 5% CO_2 atmosphere, with cleavage evaluated twice daily. After 2 days in culture, embryos that had developed to the blastocyst stage (E3.5) were analyzed for mRNA by TaqMan-qPCR using primers listed in Table S1.

Generation of *Pcbp1*-heterozygous mice

Guide RNAs (gRNAs) were designed using the Benchling online service (https://benchling.com/) and synthesized as described previously (Battulin et al., 2021). For intracytoplasmic microinjection, *in vitro*–fertilized oocytes were obtained from superovulated C57BL/6 females and spermatozoids from C57BL/6 males following established protocol (Takeo and Nakagata, 2018). Fertilized oocytes were microinjected with one of the two solutions (1) 120 ng/μL ssODN-1, 25 ng/μL sgRNA-1, 25 ng/μL sgRNA-2, and an equimolar concentration of Alt-R HiFi Cas9 Nickase V3 (IDT, Coralville, IA, USA) or (2) 120 ng/μL ssODN-2, 25 ng/μL sgRNA-4 (Table S2), and an equimolar concentration of Alt-R HiFi Cas9 Nuclease V3 (IDT, Coralville, IA, USA). After microinjection, zygotes were cultured overnight at 37°C in a 5% CO_2 atmosphere, and resulting 2-cell–stage embryos were transferred into the oviducts of pseudopregnant CD-1 females. Offspring were genotyped for target modifications using Pcbp1-gen primers (Table S2), and PCR products were sequenced by the Sanger method. Founders #6 (from experiment with Cas9-nuclease) and #19 (from experiment with Cas9-nickase) carrying loss-of-function *Pcbp1* alleles were selected and maintained through breeding with C57BL/6 mice. Heterozygous *Pcbp1* (HET) offspring were intercrossed to generate homozygous (KO), HET, and WT embryos for developmental analysis. Successful mating was confirmed by the presence of a copulation plug, and embryos were staged as embryonic day (E) 0.5 from noon on the day of the plug detection. Post-implantation embryos were dissected from the uterus, placed in PBS, photographed using the EVOS fl Auto visualization system (ThermoFisher), and genotyped using Pcbp1-gen primers (Table S2).

Cell culture work

Unless otherwise specified, all reagents for mouse ESC culturing were purchased from Gibco (ThermoFisher Scientific). ESCs were routinely cultured on adhesive plastic dishes (Eppendorf or TPP) precoated with 0.1% gelatin (Merck) under standard conditions (5% CO_2, 37°C). The mES medium consisted of Knockout-DMEM supplemented with 15% fetal bovine serum (HyClone), 1x penicillin/streptomycin, 2 mM L-Glutamine, 1x non-essential amino acids, 50 μM β-mercaptoethanol, and 500 U/mL bacterially expressed human LIF produced in-house. Naive ESC culturing and EpiLC induction were performed as described previously (Hayashi et al., 2011). ESCs were first cultured for 6–8 days on the poly-L-ornithine–coated plastic in 2i-LIF-N2B27 medium. This medium was a 1:1 mixture of N2 (DMEM/F12 + GlutaMAX with addition of N2 supplement, 1x Pen-Strep, 0.005% BSA, and 50 μM β-mercaptoethanol) and B27 (Neurobasal medium with addition of B27 supplement, 2 mM L-Gln, 1x Pen-Strep and 50 μM β-mercaptoethanol), supplemented with 500 U/mL hLIF, 3 μM CHIR99021 (Axon) medium, and 1 μM PD0325901 (Axon). ESCs were passaged using 0.05% Trypsin-EDTA and were used for EpiLC/EpiSC derivation after no more than 8 days in 2i-LIF-N2B27 culture, as prolonged MEK inhibition is known to induce genomic and epigenetic changes (Choi et al., 2017; Yagi et al., 2017). For derivation of EpiLCs, 28,000 naive ESCs per cm^2 were seeded on fibronectin (Merck)-coated (15 μg/mL) plastic in EpiLC medium: N2B27 supplemented with 12 ng/mL bFGF (Peprotech), 20 ng/mL ACTIVIN A (Peprotech), and 1% knockout serum replacement. For further maturation of EpiSCs (Guo et al., 2009; Tosolini and Jouneau, 2016), XAV939 was added from day 2 (EpiLC stage) to prevent spontaneous differentiation (Sugimoto et al., 2015; Sumi et al., 2013). Cells were passaged using collagenase IV, and 10 μM XAV939 was added to the EpiLC medium. Due to differences in proliferation rates of Scr and KO cells, KO cells were seeded at 2.3-fold the density of Scr cells on Day 0 to ensure equal cell numbers for energy profiling and proteomic, metabolomic, and protein biosynthesis assays. Cultivation in AloXR medium was performed as described (Kinoshita et al., 2020). Medium was comprised of 3 ng/mL of activin A, 2 μM XAV939 and 1.0 μM BMS493 in N2B27 medium, passaging was performed using accutase and plastic dishes were pre-treated by 15 μg/mL Fibronectin (R&D). Gene editing was performed by transfection of genetic constructs with FuGENE (Roche), selection was conducted using antibiotics G418 (500 ng/mL) for PCBP1-rescue assay and puromycin (2 μg/mL) during the T2A-EGFP integration into *Mthfd2* locus. Cell lines tested negative for mycoplasma by periodic PCR screening.

Genetic constructs

The plasmid *pX330-U6-Chimeric_BB-CBh-hSpCas9* harboring *EGFP* gene and *Rosa26*-targeting gRNA was reported previously (Kuzmin et al., 2019). The plasmid *pPcbp1-Rosa26* was established by cloning *Pcbp1* cDNA into the *pRosa26-TRE-CAG-Frt(Ert2CreErt2-STOP)Frt-tdTomato-PGKneo* construct as described (Kuzmin et al., 2019). Plasmid *CRISPR_Mthfd2-KI* for CRISPR/Cas9-mediated break within *Mthfd2* 3′-end for *T2A-EGFP* integration was established by incorporation

gRNA-sequences (Table S3) into *lentiCRISPR v2* cloning vector (Addgene #52961) by BsmbI restriction sites. Donor vector harboring *T2A-EGFP* with 100-bp homology arms to *Mthfd2* locus flanked by *Mthfd2-KI* gRNA sequences was designed as described (Zhang et al., 2017) and ordered from Evrogen (Moscow, Russia). ShRNA for *Atf4* knockdown (Table S3) was cloned into pLKO.1 cloning vector (Addgene #10878) according to the manufacturer's recommendations.

Cell death analysis

Propidium iodide (PI) or DAPI were added directly to the medium at a final concentration of 50 μg/mL for 2 min. The medium containing PI and floating cells was collected and combined with cells harvested from the well using Trypsin/EDTA. Cells were centrifuged, washed, and resuspended in PBS before being analyzed by FACS. Alexa Fluor 647–labeled annexin V antibodies were used according to the manufacturer's recommendations.

RNA extraction and qPCR analysis

Total RNA was extracted using Extract RNA reagent (Evrogen, Moscow, Russia) according to manufacturer's instructions and treated with DNaseI (ThermoFisher). The quality and concentration of extracted RNA samples were measured using a NanoDrop 2000 spectrophotometer (ThermoFisher). cDNA was synthesized using an MMLV RT kit (Evrogen) with random primers. The quantitative polymerase chain reactions (qPCR) were performed with 5X qPCRmix-HS SYBR+Low-ROX (Evrogen) and gene-specific primers (Table S4) on an Applied Biosystems 7300 Real-Time PCR System. The relative quantification of target genes was calculated using the $2-\Delta\Delta Ct$ method with the *Gapdh* used as the reference gene. The absence of nonspecific amplification and dimer formation of primers was checked by setting negative controls without a cDNA template and analyzing the melting curves of the amplification products.

Immunocytochemical analysis

Cells were fixed in 4% paraformaldehyde (Merck) for 10 min at room temperature (RT), permeabilized with 0.1% Triton X-100 for 15 min, and blocked in 3% BSA for 1 h at RT. Primary antibody staining was performed overnight at 4°C using the appropriate antibodies (see key resources table) diluted in PBS containing 0.1% Tween 20 (PBST). Cells were then washed 5–6 times with PBST, stained with secondary fluorescent antibodies (Jackson ImmunoResearch) for 2 h at RT, and washed 2 or 3 additional times with PBST. Nuclei were stained with DAPI in PBST for 5 min at RT, and cells were stored in PBS containing NaN_3 until imaging. Fluorescent microscopy was performed using an EVOS FL AUTO (Life Technologies) microscope equipped with DAPI, GFP, RFP, and CY5 filter cubes.

Western blot

For Western blot analysis, cells were harvested and resuspended in PBS. An 1/10 aliquot was taken for protein concentration measurement using the Pierce BCA Protein Assay Kit (ThermoFisher Scientific). Then, 2x Laemmli buffer containing β-mercaptoethanol was added, and probes were boiled at 100°C for 5 min. For electrophoresis, 10–20 μg of total protein from each probe was loaded onto a 10% acrylamide gel and separated at 80 V. Proteins were transferred onto a nitrocellulose membrane (Bio-Rad), which was then blocked with 5% non-fat milk in PBST. Incubation with primary antibodies was performed in 3% BSA-PBST for 2 h at RT. After 3 or 4 washes in PBST, membranes were incubated with secondary antibodies diluted in 1% fat-free milk in PBST for 1 h, then washed three times in PBST. Chemiluminescence was visualized using the SuperSignal West Pico PLUS Chemiluminescent Substrate (ThermoFisher Scientific) and visualized using the ChemiDoc Touch Imaging System (Bio-Rad).

In vitro differentiation

Mesodermal and endodermal differentiation was proceeded via embryoid bodies (EB). To this end, 600 Scr or 1200 KO ESCs in 35-μL hanging drops of mES-medium (without LIF) were placed on the Petri dish cover and incubated for five days. Next, EB were transferred to gelatin-coated plastic dishes and cultured for additional 10 days in DMEM/F12 + GlutaMAX supplemented with 10% FBS and 1x Pen-Strep. For neuroectodermal differentiation, Scr and KO EpiSC clumps were plated on fibronectin-coated plastic in N2B27 for three days, then medium was changed to DMEM/F12 + GlutaMAX supplemented with 10% FBS and 1x Pen-Strep for additional three days. After differentiation cells were fixed and subjected to immunocytochemical analysis.

Cell cycle analysis

Cells were harvested by trypsinization, washed with PBS, and resuspended in 100 μL PBS supplemented with 0.025% saponin, 50 μg/mL PI, and 0.25 mg/mL RNAse A for 40 min at RT. The cells were then analyzed by flow cytometry, and cell cycle distribution was assessed using ModFit LT 3.0 software (Verity Software House, Lexington, MA, USA).

Time-lapse microscopy

The naïve-to-primed transition was performed as described above except that cells were seeded at a low density (100 cells/cm^2) for 4 h in the 2i-LIF-N2B27 medium. Then, the medium was replaced with EpiLC medium, and time-lapse microscopy was performed using the CQ1 confocal system (Yokogawa).

Flow cytometry

Flow cytometry was performed using a CytoFLEX benchtop flow cytometer (Beckman Coulter) with laser wavelengths of 585/42 nm for PI detection (PE) and 660/20 nm for Alexa 647-conjugated secondary antibody detection (APC).

SeaHorse energy profiling

Mitochondrial and glycolytic energy metabolism was assessed using a Seahorse XFe24 Analyzer (Agilent, Santa Clara, CA, USA) and the Seahorse XF Real-Time ATP Rate Assay Kit (Agilent, Santa Clara, CA, USA) according to the manufacturer's instructions. Cells were seeded in 24-well SeaHorse plates in quadruplicate. Oligomycin and the Rotenone/Antimycin A mixture were added at final concentrations of 3 μM. GlycoATP and mitoATP production were quantified using the SeaHorse XF Real-Time ATP Rate Assay Excel plugin (Agilent, Santa Clara, CA, USA). Data are presented as means ± SEM.

Quantitative comparison of total protein biosynthesis

The intensity of total protein biosynthesis was quantified using the Protein Synthesis Assay Kit (ab239725) with flow cytometry, following the manufacturer's instructions. Results are expressed as the means ± SD.

Surface sensing of translation (SUnSET) assay

The SUnSET assay was performed as described elsewhere (Goodman and Hornberger, 2013). Briefly, ESCs were seeded in 3-cm cell culture dishes either in 2i-LIF-N2B27 or EpiLC medium two days prior to the assay. The cells were then exposed to 10 μg/mL puromycin for 15 min. During this period, puromycin, a tyrosyl-tRNA analog, was incorporated into growing polypeptide chains, providing a real-time view of translation levels inside the cells. Following treatment, the cells were harvested and subjected to immunoblotting with anti-puromycin and anti–β-actin antibodies.

ChIP-seq analysis

Cells were fixed directly on 6-cm culture dishes with 1% formaldehyde for 10 min, quenched with 0.125 M Glycine for 5 min, washed in PBS, scraped, and resuspended in sonication buffer (50 mM HEPES-KOH, pH 7.9, 140 mM NaCl, 1 mM EDTA, 1% Triton X-100, 0.1% Na deoxycholate, 0.1% SDS) containing a protease inhibitor cocktail (PIC, Roche). Chromatin was then fragmented using a CL-18 sonicator (ThermoFisher Scientific) for 10 s with 60% amplitude, repeated 10 times with 60-s cooling on ice after each cycle. Afterward, immunoprecipitation was performed as follows: chromatin from 1.5×10^6 ESCs or EpiLCs was incubated with 12 μg of antibodies to PCBP1, conjugated with MabSelect sure Sepharose (Cytiva), preliminary blocked with 1% BSA. Precipitation was performed in presence of 1% BSA with rotation for 1.5 h at +4°C. Sepharose was washed twice with sonication buffer and once with TE buffer. Then precipitated IgG-protein-DNA complexes were eluted by elution buffer (50 mM Tris-HCl, pH 8.0, 1 mM EDTA, 1% SDS) for 30 min with rotation at room tempreture, and crosslinking was reversed by incubation with proteinase K, NaCl, and EDTA at 55°C for 10 h and at 65°C for 5 h. DNA was purified using phenol:chloroform extraction, dissolved in 50 μL of TE buffer, and used for DNA library preparation and NGS sequencing.

DNA libraries were prepared for sequencing using standard Illumina protocols. The libraries were sequenced to generate high-quality paired-end reads. Raw sequencing reads were processed using fastp (Chen, 2023) to trim adapters and remove low-quality bases with default parameters. The cleaned reads were aligned to the GRCm38/mm10 mouse reference genome using Bowtie2 (Langmead and Salzberg, 2012) with default settings. Duplicate reads were identified and marked using Picard MarkDuplicates (http://broadinstitute.github.io/picard/). Peak calling was performed using MACS2 (Liu, 2014) with a stringent false discovery rate threshold (q) < 0.01, using matched input DNA as a control for each corresponding sample. To generate BigWig files for visualization, MACS2 bdgcmp was used to subtract the bedgraph pileup of the treatment sample from the input control. Peak annotation and enrichment analysis were conducted using the

ChIPseeker R package (Wang et al., 2022) to identify genomic features associated with the called peaks and to gain insights into the biological relevance of the results. Integrated genome browser (Freese et al., 2016) was used for peak visualization.

RNA-seq analysis

Total RNA was extracted using TRIzol (Invitrogen) following the manufacturer's recommendations. NGS libraries were constructed using the TruSeq Stranded mRNA Kit (Illumina) following the manufacturer's protocol. 1 μg of total RNA was used as the input material for the protocol. RNA extraction, library construction, and sequencing were performed by Evrogen JSC (Moscow, Russia). RNA-seq libraries were sequenced to generate single-end reads. Raw sequencing reads were processed using fastp (Chen, 2023) to remove adapter sequences and low-quality bases, applying default parameters. Quality control of the cleaned reads was assessed using FastQC (Andrews, 2010). High-quality reads were mapped to the GRCm38/mm10 mouse reference genome using the STAR aligner (Dobin et al., 2013) in two-pass mode to improve splice junction detection. Further quality control of the aligned reads, including read distribution and gene body coverage, was performed using RSeQC tools (Wang et al., 2012). Gene-level read counts were quantified using featureCounts, and differential expression analysis between the knockout (KO) and wild-type (Scr) groups was conducted with DESeq2 (Love et al., 2014). Gene Ontology (GO) enrichment analysis was performed using the FGSEA method, implemented in the clusterProfiler R package (Wu et al., 2021), to identify enriched biological processes and pathways associated with differentially expressed genes.

Shotgun proteomics

Cells were washed with PBS and lysed in RIPA buffer (ThermoFisher) supplemented with protease (Roche) and phosphatase inhibitors (Sigma). The samples were subjected to freeze-thaw cycles, sonicated in an ultrasonic bath, and proteins were cleaned from lysis buffer components by acetone precipitation. The protein pellet was resuspended in 8M Urea/50 mM ammonium bicarbonate, and the protein concentration was measured using a Qubit fluorometer (ThermoFisher) with the QuDye Protein Quantification Kit (Lumiprobe) according to the manufacturer's recommendations. The samples (20 μg) were incubated for 1 h at 37°C in 5 mM DTT (Sigma), followed by incubation in 15 mM iodoacetamide for 30 min in the dark at room temperature (Sigma). The samples were then diluted with seven volumes of 50 mM ammonium bicarbonate and incubated for 16 h at 37°C with 400 ng of trypsin (ratio 1:50; Promega). Finally, the samples were evaporated in Eppendorf Concentrator Plus (Eppendorf), dissolved in water ("LC-MS" grade; LiChrosolv) with 0.1% formic acid ("for LC-MS LiChropur"; Merck), and desalted using C18 Zip Tips (Merck) according to manufacturer's recommendations. Desalted peptides were evaporated and dissolved in water/0.1% formic acid for further LC-MS/MS analysis.

Approximately 1 μg of each sample was used for shotgun proteomics analysis by HPLC-MS/MS with ion mobility, using a TimsToF Pro mass spectrometer (Bruker Daltonics, Bremen, Germany) coupled with nanoElute UHPLC chromatography (Bruker Daltonics, Bremen, Germany). UHPLC was performed in the two-column separation mode with an Acclaim PepMap 5-mm Trap Cartridge (Thermo Fisher Scientific, Waltham, MA, USA) and an Aurora Series separation column with nanoZero technology (C18, 25 cm × 75 μm ID, 1.6 μm, C18) in gradient mode at a flow rate of 400 nL/min and a column temperature of 40°C. Phase A was water/0.1% formic acid, and phase B was acetonitrile/0.1% formic acid (LC-MS Grade). The gradient was from 2% to 35% phase B for 40 min, then to 85% phase B for 5 min, followed by a wash with 85% phase B for 10 min. The column was equilibrated with 4 column volumes before each sample. Electrospray ionization was performed using the CaptiveSpray ion source with a capillary voltage of 1600 V, a 3 L/min N2 flow, and a source temperature of 180°C. Mass spectrometry acquisition was performed in automatic DDA PASEF mode with a 0.5-s cycle in positive polarity, fragmenting ions with at least two charges within the m/z range of 100–1700 and an ion mobility range of 0.85–1.30 1/K0. Each sample was analyzed in at least two analytical replicates.

Protein identification was performed using *Mus musculus* proteins available in UniProt (downloaded 25.02.2022, 34162 proteins), including contaminations. DDA-PASEF data analysis was carried out in FragPipe (v. 17.1) according to the default LFQ-MBR workflow (Kong et al., 2017). The search parameters consisted of a parent and fragment mass error tolerance of 10 ppm, a protein and peptide FDR of less than 1%, trypsin as the protease (cleaving after KR, no cleavage before P), and two possible missed cleavage sites. Cysteine carbamidomethylation was set as a fixed modification, while methionine oxidation, STY phosphorylation, and acetylation of protein N-termini were set as variable modifications. Data filtration and imputation of missing values were performed using the NAguideR package for proteomic data analysis (Wang et al., 2020). Proteins with missing values in more than half of the samples in at least one biological group and proteins with a coefficient of variation greater than 0.6 were discarded. The optimal method for missing value imputation was selected

using “classic criteria,” specifically the “Robust data imputation” approach (“Impseqrob”) (Branden and Verboven, 2009). Differential expression analysis was performed by “limma” with Log2-transformed data normalized by quantile normalization (Ritchie et al., 2015).

Metabolomics

Cells cultured in 6-well plates were washed twice with ice-cold PBS, covered with 500 μL of pre-chilled methanol (−80°C), scraped off the plastic, and transferred to Eppendorf tubes. An additional 100 μL of methanol was used to collect the remaining cells. The samples were vortexed and centrifuged for 15 min at +4°C. The supernatant (450 μL) was transferred into a new tube for metabolite analysis, while the residual material was used for total protein measurement for subsequent normalization. For derivation of amino acids, 100 μL of the methanol-based total metabolome extract was dried under vacuum (Concentrator Plus; Eppendorf, Germany) at 30°C. The resulting pellet was resuspended in 30 μL of 1-butanol containing 3M hydrochloric acid and incubated at 62°C for 20 min with gentle stirring. After cooling for 1–2 min, the reaction was stopped by adding 30 μL of deionized water. The samples were dried under vacuum at 30°C, and the resulting pellet was reconstituted in 200 μL of 0.1% formic acid. For derivation of carboxylic acids, 100 μL of the methanol-based total metabolome extract was dried under vacuum at 30°C. The resulting pellet was resuspended in 15 μL of 10 mM triphenylphosphine in acetonitrile to activate carboxylic acids into acyloxyphosphines. Following activation, 15 μL of 10 mM 2-pycolilamine in acetonitrile and 15 μL of 10 mM 2.2′-dithiodipyridine in acetonitrile were added. The reaction mixture was incubated for 20 min at 60°C with constant gentle stirring. After the reaction was completed, the samples were cooled down, and the reaction was stopped by adding 45 μL of deionized water. Samples were dried under vacuum at 30°C, and the resulting pellet was reconstituted in 50 μL of 0.1% formic acid.

The analysis was performed using a G6490A triple quadrupole mass spectrometer (Agilent, Inc., Santa Clara, CA, USA). Detection and quantification of amino acids was performed in dynamic SRM (selected reaction monitoring) mode, while carboxylic acids were detected using dynamic neutral loss-SRM (NL-SRM) mode by registering and recording the signal of the 2-pycolylamine–derived product ion (m/z = 109.076). The samples were separated using the UPLC Infinity II 1290 chromatography system (Agilent, Inc., Santa Clara, CA, USA). Samples containing amino acids were loaded at a volume of 5 μL onto the Zorbax Eclipse Plus C18 RRHD column (2.1 × 50 mm, 1.8-μm particle size; Agilent, Inc, Santa Clara, CA, USA) and separated at a flow rate of 0.3 mL/min in a stepwise gradient of mobile phases A (water) and B (acetonitrile). Samples with carboxylic acids were loaded in a volume of 10 μL and separated at a flow rate of 0.3 mL/min on an Acquity Premier HSS T3 column (2.1 × 150 mm, 1.8 μm particle size; Waters Inc, Ireland) in a gradient of mobile phases A (water) and B (acetonitrile).

QUANTIFICATION AND STATISTICAL ANALYSIS

Statistical analysis was performed using GraphPad Prism. Unless otherwise specified, data were analyzed using an unpaired Student’s *t* test. The significance levels were as follows: ns, not significant; $*p < 0.05$; $**p < 0.01$; $***p < 0.001$; $****p < 0.0001$. Error bars indicate SD of the mean.

Stem Cell Reports, Volume *21*

Supplemental Information

PCBP1 orchestrates amino acid metabolism burst during the naïve-to-primed pluripotency transition

Evgeny I. Bakhmet, Evgeniy V. Potapenko, Oleg Y. Shuvalov, Arseniy A. Lobov, Egor A. Repkin, Daria V. Kriger, Nadezhda E. Vorobyeva, Alexey N. Korablev, Anna S. Zinovyeva, Andrey A. Kuzmin, Nikolay D. Aksenov, Arthur T. Kopylov, Guangming Wu, Hans R. Schöler, and Alexey N. Tomilin

A

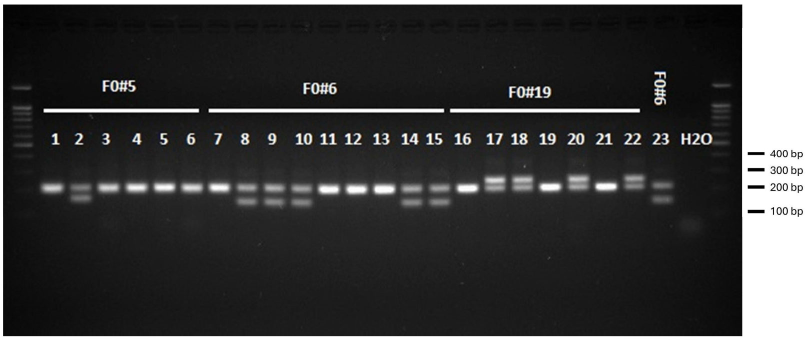

B

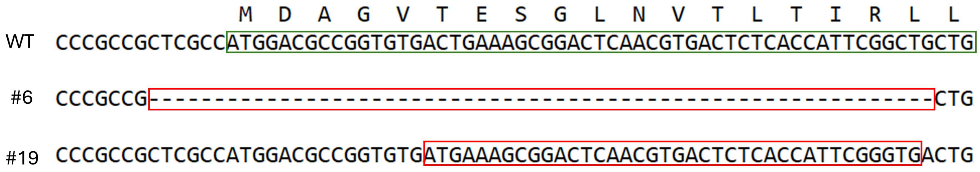

C

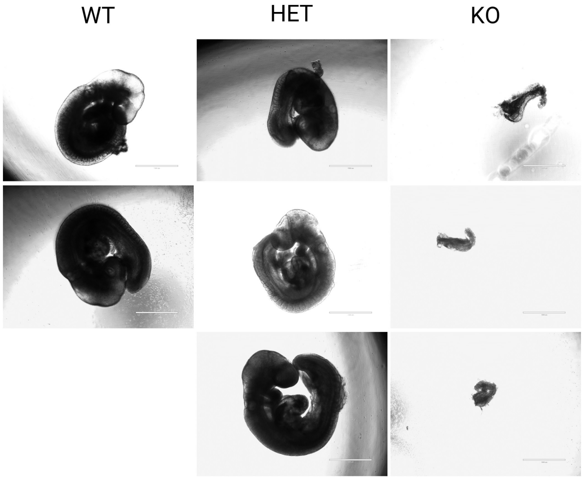

Stage	Decidua	WT	HET	KO
E6.5	7	2	2	2
E7.5	10	2	4	3
E8.5	11	2	6	2
E9.5	10	2	5	3

Figure S1. Heterozygous *Pcbp1*-null embryos characterization

(A) Genotyping of the F0 offspring reveals wild-type and heterozygous pups with deletions (#5 and #6) and an insertion #19 at the start of the protein-coding sequence of the *Pcbp1* gene. F1 offspring from the #6 and #19 founders were selected for further analysis and mice colony propagation. (B) Alignments showing affected alleles of the #6 (60 bp deletion) and #19 (38 bp insertion) lines. (C) Left panel: microphotographs of E9.5 embryos after intercrossing of *Pcbp1*-heterozygous mice. Scale bar: 1000 μm. Right panel: table representing number of decidua and recovered embryos at different developmental stages.

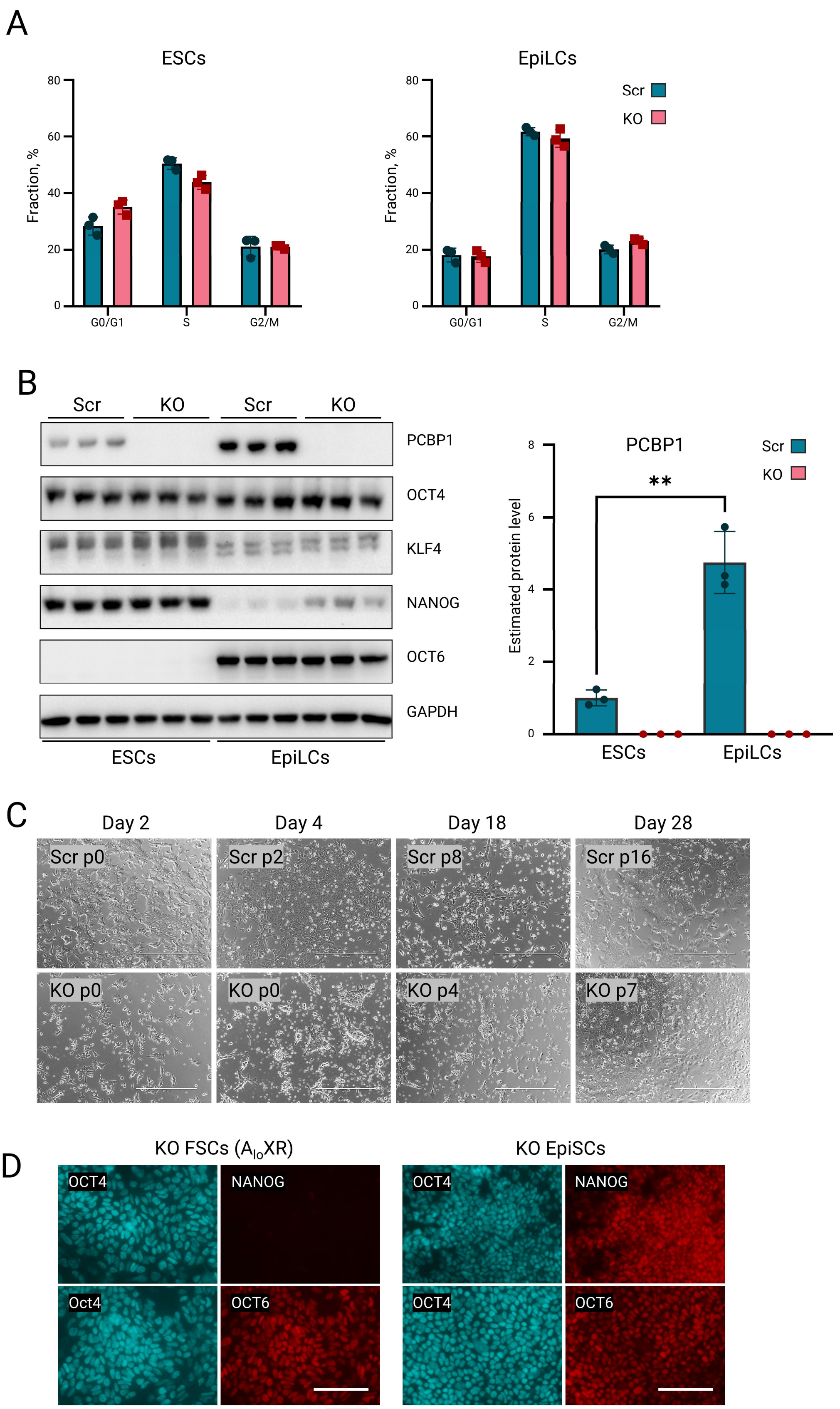

Figure S2. Naïve-to-Primed pluripotency transition of the KO ESCs

(A) Cell cycle analysis of Scr and KO cells in the naïve (ESC) and formative (EpiLC) states of pluripotency. N = 3 biological replicates (individual cell clones); error bars indicate SD of the mean. (B) Left panel: western blot

analysis of Scr and KO cells in ESC and EpiLC states for pluripotency markers. Right panel: quantification analysis of PCBP1 amount from western blot. N = 3 biological replicates (individual cell clones). Error bars indicate SD of the mean. ** $p < 0.01$. (C) Microphotographs of Scr and KO cells passaged in formative $A_{lo}XR$ culture medium. Scale bar: 400 μm. (D) Immunofluorescence microscopy of *Pcbp1*-KO cells after passaging for 28 days in $A_{lo}XR$ medium. Scale bar: 100 μm.

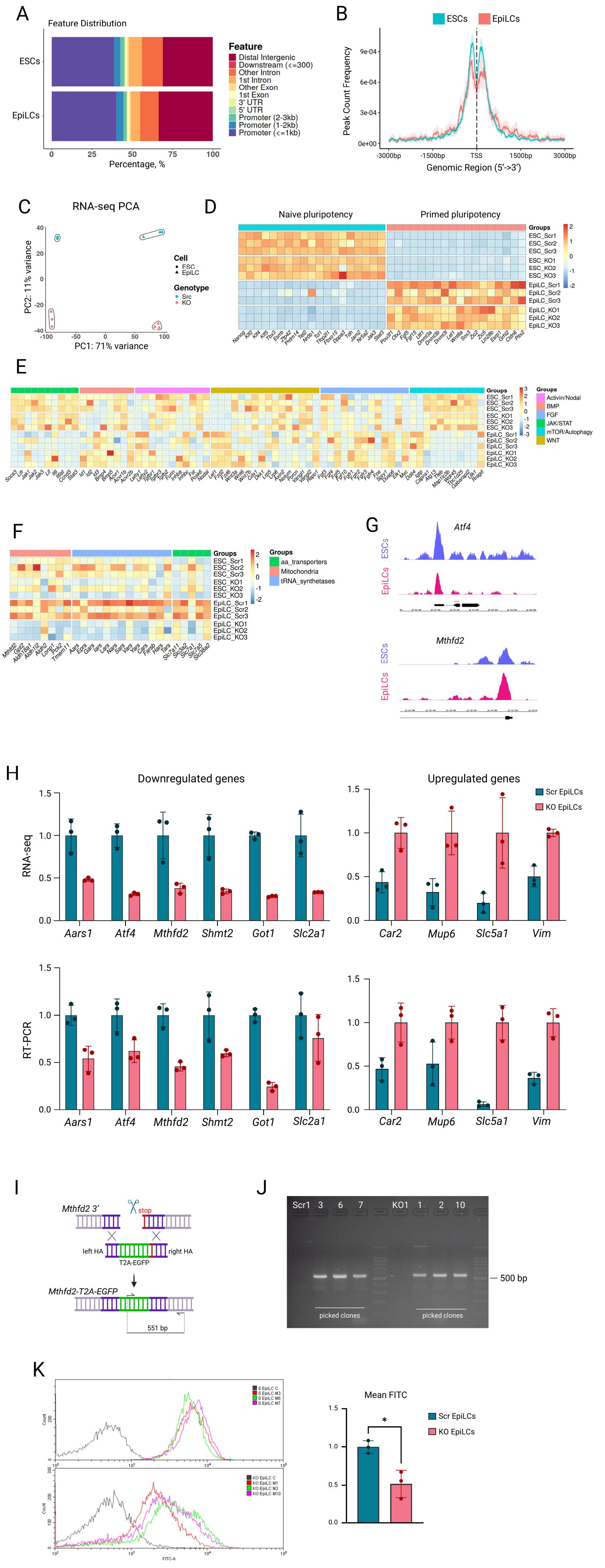

A
Feature Distribution
ESCs
EpiLCs
Percentage, %
Feature
Distal Intergenic
Downstream (<=300)
Other Intron
1st Intron
Other Exon
1st Exon
3' UTR
5' UTR
Promoter (2-3kb)
Promoter (1-2kb)
Promoter (<=1kb)
B
ESCs
EpiLCs
Peak Count Frequency
Genomic Region (5'->3')
C
RNA-seq PCA
PC1: 71% variance
PC2: 11% variance
Cell
ESC
EpiLC
Genotype
Src
KO
D
Naive pluripotency
Primed pluripotency
Groups
E
Activin/Nodal
BMP
FGF
JAK/STAT
mTOR/Autophagy
WNT
F
aa_transporters
Mitochondria
tRNA_synthetases
G
Atf4
Mthfd2
ESCs
EpiLCs
H
Downregulated genes
Upregulated genes
RNA-seq
RT-PCR
Scr EpiLCs
KO EpiLCs
Aars1
Atf4
Mthfd2
Shmt2
Got1
Slc2a1
Car2
Mup6
Slc5a1
Vim
I
Mthfd2 3'
stop
left HA
right HA
T2A-EGFP
Mthfd2-T2A-EGFP
551 bp
J
Scr1
3
6
7
KO1
1
2
10
500 bp
picked clones
K
Mean FITC
Scr EpiLCs
KO EpiLCs

Figure S3. Omics assay reveals PCBP1 function in amino acid metabolism induction

(A) PCBP1 binding site distribution in ESC and EpiLC genomes revealed by ChIP-seq data analysis. (B) PCBP1 binding site distribution around transcription start sites (TSS). (C) Principal component analysis showing clustering of RNA-seq samples by Scr vs. KO and ESC vs. EpiLC. (D) Heatmap displaying gene expression profiles related to naïve and primed pluripotency in Scr and KO cells (N = 3 biological replicates, i.e. individual cell clones). (E) Heatmap showing expression of genes related to different signaling pathways typical for pluripotent stem cells. (F) Heatmap showing expression of typical targets of the transcription factor ATF4. (G) Integrated genome browser snapshots of ChIP-seq data showing examples of genes occupied by PCBP1 in ESCs and EpiLCs. (H) Validation of the RNA-seq results by RT-PCR. N = 3 biological replicates (individual clones). Error bars indicate SD of the mean. (I) Schematic representation of the *T2A-EGFP* transgene knocked into the *Mthfd2* locus (generated using biorender.com). (J) Genotyping of picked clones for the *T2A-EGFP* insertion. Parental Scr1 and KO1 clones were used as controls. (K) Left panel: flow cytometry analysis comparing EGFP signal among the *Mthfd2-T2A-EGFP* clones in the EpiLC state. Right panel: quantification of EGFP signal presented in the left panel. N = 3 biological replicates (individual cell clones). Error bars indicate SD of the mean. * $p < 0.05$.

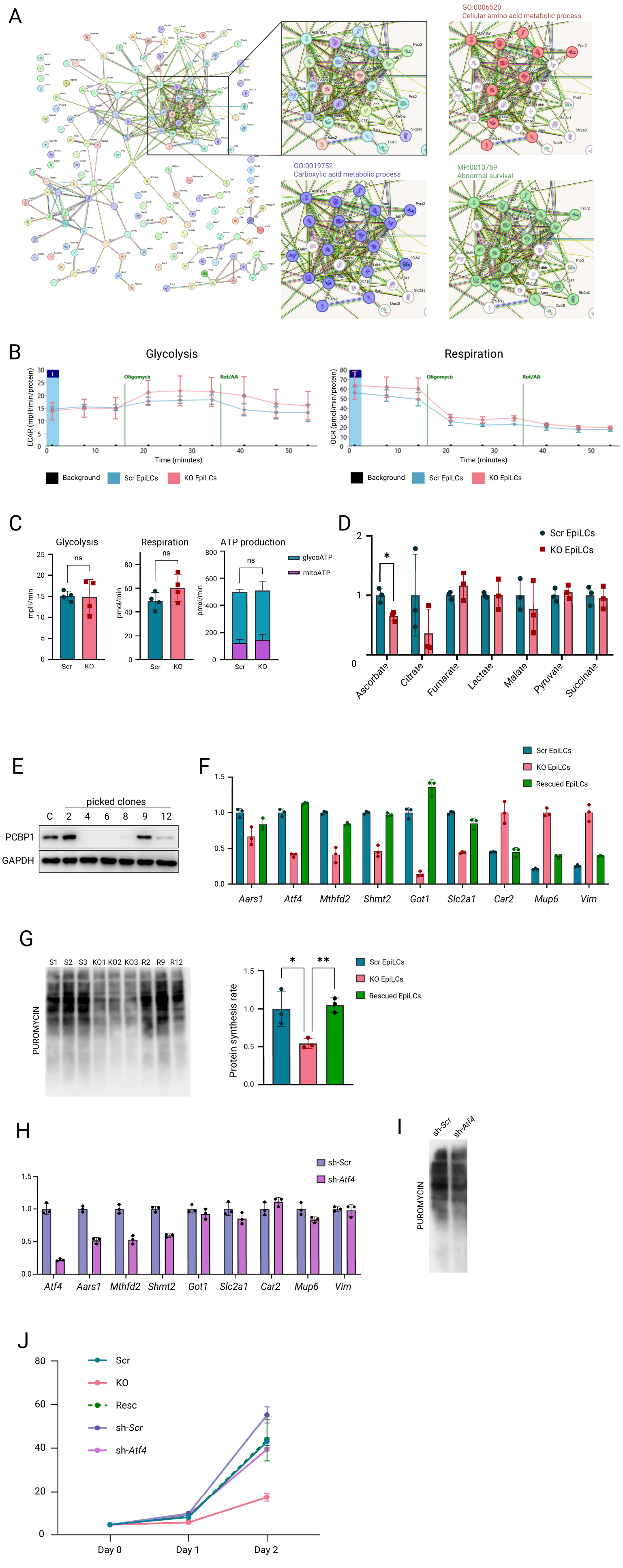
A
GO:0006520
Cellular amino acid metabolic process
GO:0019752
Carboxylic acid metabolic process
MP:0010769
Abnormal survival
B
Glycolysis
Respiration
ECAR (mpH/min/protein)
OCR (pmol/min/protein)
Oligomycin
Rot/AA
Time (minutes)
Background
Scr EpiLCs
KO EpiLCs
C
Glycolysis
Respiration
ATP production
mpH/min
pmol/min
ns
glycoATP
mitoATP
Scr
KO
D
Scr EpiLCs
KO EpiLCs
Ascorbate
Citrate
Fumarate
Lactate
Malate
Pyruvate
Succinate
E
picked clones
C 2 4 6 8 9 12
PCBP1
GAPDH
F
Scr EpiLCs
KO EpiLCs
Rescued EpiLCs
Aars1
Atf4
Mthfd2
Shmt2
Got1
Slc2a1
Car2
Mup6
Vim
G
S1 S2 S3 KO1 KO2 KO3 R2 R9 R12
PUROMYCIN
Protein synthesis rate
Scr EpiLCs
KO EpiLCs
Rescued EpiLCs
H
sh-Scr
sh-Atf4
Atf4
Aars1
Mthfd2
Shmt2
Got1
Slc2a1
Car2
Mup6
Vim
I
sh-Scr
sh-Atf4
PUROMYCIN
J
Scr
KO
Resc
sh-Scr
sh-Atf4
Day 0
Day 1
Day 2

Figure S4. PCBP1 is crucial for protein synthesis in priming pluripotent cells

(A) Clustering of differentially abundant proteins in EpiLCs using STRING (string-db.org), with several enriched pathways highlighted. (B) ECAR (glycolysis) and OCR (respiration) measurements using the SeaHorse XFe96 analyzer. (C) Energy metabolism of Scr and KO EpiLCs using a SeaHorse analyzer, measuring glycolysis (ECAR), respiration (OCR), and calculated ATP production. N = 4 technical replicates. Error bars indicate SD of the mean. (D) Metabolome analysis of carboxylic acids in Scr and KO EpiLCs. N = 3 biological replicates (individual clones). Error bars indicate SD of the mean. *p<0.05. (E) Western blot analysis of the picked clones for PCBP1 after knocking exogeneous *Pcbp1* into the *Rosa26* locus. (F) Representative results of RT-PCR analysis aimed at verification of restoration of gene expression in *Pcbp1*-rescued EpiLCs. n = 3 technical replicates. Error bars indicate SD of the mean. (G) Left panel: SUnSET assay showing restoration of the protein synthesis rates in *Pcbp1*-rescued EpiLCs. Right panel: quantification analysis of Puromycin signal presented on the left panel. N = 3 biological replicates (individual clones). Error bars indicate SD of the mean.; *p<0.05; **p<0.01. (H) RT-PCR analysis of control EpiLCs (shScr) and with shRNA-mediated *Atf4* knockdown (shAtf4). n = 3 technical replicates. Error bars indicate SD of the mean. (I) SUnSET assay showing protein synthesis rate in control EpiLCs (sh-*Scr*) and with *Atf4* knockdown (sh-*Atf4*). (J) Proliferation rates of Scr, KO, Rescued, sh-*Scr*, and sh-*Atf4* cells during the naïve-to-primed pluripotency transition. For Scr, KO, and Resc N = 3 biological replicates (individual clones); for sh-*Scr* and sh-*Atf4* n=3 (technical replicates); error bars indicate SD of the mean.

Supplemental Tables

Table S1. Oligonucleotides used for *Pcbp1* knockdown and analyses in pre-implantation embryos

Control siRNA	GCACCCGATAAGCGGTCAA
Pcbp1 siRNA-1	GTGTGACTGAAAGCGGACTCA
Pcbp1 siRNA-3	GAACCAGGTGGCAAGACAA
Pcbp1 siRNA-5	GCTGATGCACGGAAAGGAA
Hprt1	Mm00446968_m1
Pou5f1	Mm00658129_gH
Pcbp1	Mm00478712_s1
5' *GFP*	CCACATGAAGCAGCACGACT
3' *GFP*	TGCGCTCCTGGACGTAGC
GFP Probe	6-FAM-TTCAAGTCCGCCATGCCCGAA-TAMRA

Table S2. Oligonucleotides used for generation and analysis of *Pcbp1*-heterozygous mice

gRNA#1 (nickase)	gTCGGCTGCTGATGCACGGAA (AGG)
gRNA#2 (nickase)	gACACCGGCGTCCATGGCGAG (CGG)
ssODN-1 (nickase)	CCTCGCGGATCCTCTTCACCGACTCCCCTTTCTTCCCGATGATGCTGCCTACTTCCTTTCGAGCGGCGGGCGGCGTTCGGGAGTTGGGCTCGTTACGTGGTCAAGTCCTGGGCGGCTGGC
gRNA#3 (nuclease)	gTCAACGTGACTCTCACCATT (CGG)
ssODN-2 (nuclease)	ATGATGCTGCCTACTTCCTTTCCGTGCATCAGCAG**g**CGAATGGTGAGAGTCACGTTGAGTCCGCTTTCAGT
Pcbp1-gen_F	ACTTGACCACGTAACGAGCC
Pcbp1-gen_R	CCCCTCCGAGATGTTGATCC

Table S3. CRISPR/Cas9 and knockdown in ESCs

sh-*Scr* (Addgene #1864)	CCTAAGGTTAAGTCGCCCTCGCTCGAGCGAGGGC GACTTAACCTTAGG
sh-*Atf4* (Zhao et al., 2025)	CCGGCCATCTCCCAGAAAGTTTAATCTCGAGATTA AACTTTCTGGGAGATGGTTTTTG
CRISPR-gRNA_*Mthfd2*-KI_F	caccgCTCAGAGTGCTGCTAGTTGG
CRISPR-gRNA_*Mthfd2*-KI_R	aaacCCAACTAGCAGCACTCTGAGc

Table S4. Oligonucleotides used in RT-PCR

Atf4_F	GATGGGTTCTCCAGCGACAAG
Atf4_R	CCGGAAAAGGCATCCTCCTT
Mthfd2_F	TCCTTGTTGTCTGCGTTGGC
Mthfd2_R	CTTCATTTCGCACTGCCGCC
Aars1_F	TTGCTATTCCCTCGGAGCAC
Aars1_R	CTCCTCGGGAACCTTAGCTC
Got1_F	AAGCAGATCGCTGCTGTCAT
Got1_R	AATAGCGAATAGCCCACGCA
Shmt2_F	AGTACGAACCGTAGACCCCA
Shmt2_R	GTACTCGCGGAACATAGGGG
Slc2a1_F	ACCTCTTCCGAACCGACAGAT
Slc2a1_R	TCTGGAGCCATCAAAGTCCTG
Vim_F	CGGCTGCGAGAGAAATTGC
Vim_R	CCACTTTCCGTTCAAGGTCAAG
Car2_F	CTCTGCTGGAATGTGTGACCTG
Car2_R	CCAGTTGTCCACCATCGCTTCT
Mup6_F	GGAAACCTTCCAGCTGATGTCG
Mup6_R	CTCTAATGATTCCATGCTCCTCAC
Gapdh_F	AGGTCGGTGTGAACGGATTTG
Gapdh_R	TGTAGACCATGTAGTTGAGGTCA

Zhao, L., Chen, Y., Ding, X., Li, H., and Li, J. (2025). Targeting Atf4 for enhanced neuroprotection: Role of quercetin-loaded EVs in ischemic stroke. J Pharm Anal *15*, 101312. 10.1016/j.jpha.2025.101312.
